# Mouse Mast Cell Protease 4 Deletion Protects Heart Function and Survival After Permanent Myocardial Infarction

**DOI:** 10.3389/fphar.2018.00868

**Published:** 2018-08-31

**Authors:** Martin Houde, Adel Schwertani, Hanène Touil, Louisane Desbiens, Otman Sarrhini, Roger Lecomte, Martin Lepage, Hugo Gagnon, Shinji Takai, Gunnar Pejler, Danielle Jacques, Fernand Gobeil, Robert Day, Pedro D’Orléans-Juste

**Affiliations:** ^1^Department of Pharmacology-Physiology, Faculté de Médecine et des Sciences de la Santé, Université de Sherbrooke, Sherbrooke, QC, Canada; ^2^Division of BioTherapeutics, Leiden Academic Centre for Drug Research, Universiteit Leiden, Leiden, Netherlands; ^3^Department of Medicine, McGill University, Montreal, QC, Canada; ^4^Department of Nuclear Medicine and Radiobiology, Sherbrooke Molecular Imaging Center, CRCHUS, Université de Sherbrooke, Sherbrooke, QC, Canada; ^5^PhenoSwitch Bioscience Inc., Sherbrooke, QC, Canada; ^6^Department of Innovative Medicine, Osaka Medical College, Osaka, Japan; ^7^Department of Medical Biochemistry and Microbiology, Uppsala University, Uppsala, Sweden; ^8^Department of Anatomy, Physiology and Biochemistry, Swedish University of Agricultural Sciences, Uppsala, Sweden; ^9^Department of Anatomy and Cell Biology, Faculté de Médecine et des Sciences de la Santé, Université de Sherbrooke, Sherbrooke, QC, Canada; ^10^Department of Surgery, Faculté de Médecine et des Sciences de la Santé, Université de Sherbrooke, Sherbrooke, QC, Canada

**Keywords:** chymase, mouse mast cell protease 4, myocardial infarction, positron emission tomography, glycoproteomics, active caspase-3, collagen

## Abstract

Chymase, a mast cell serine protease involved in the generation of multiple cardiovascular factors, such as angiotensin II and endothelin-1 (ET-1), is elevated and participates in tissue degeneration after permanent myocardial infarction (PMI). Anesthetized 4-month old male wild-type (WT) C57BL/6J mice and mouse mast cell protease-4 knockout (mMCP-4 KO) congeners were subjected to ligation of the left anterior descending (LAD) coronary artery. A group of mice was then subjected to Kaplan-Meier 28-day survival analysis. In another group of mice, ^18^F-fluorodeoxyglucose positron emission tomography (PET) was performed to evaluate heart function and the infarcted zone 3 days post-PMI surgery. Cardiac morphology following PMI was evaluated on formalin-fixed heart slices and glycoproteomic analysis was performed using mass spectrometry. Finally, cardiac and lung tissue content of immunoreactive ET-1 was determined. PMI caused 60% mortality in WT mice, due to left ventricular wall rupture, and 7% in mMCP-4 KO mice. Cardiac PET analysis revealed a significant reduction in left ventricular volume (systolic and diastolic) and preserved the ejection fraction in mMCP-4 KO compared to WT animals. The infarcted area, apoptotic signaling and wall remodeling were significantly decreased in mMCP-4 KO mice compared to their WT congeners, while collagen deposition was increased. Glycoproteomic analysis showed an increase in apolipoprotein A1, an established chymase substrate in mMCP-4 KO mice compared to WT mice post-PMI. ET-1 levels were increased in the lungs of WT, but not mMCP-4 KO mice, 24 h post-PMI. Thus, the genetic deletion of mMCP-4 improved survival and heart function post-PMI.

## Introduction

Myocardial remodeling and fibrosis after heart failure are associated with increased mast cell density, and mast-cell deficient mice show an improved prognosis following myocardial infarction (Levick et al., 2011). Pharmacological stabilization of mast cells also reduces the impairment of heart function in animal models (Zhang et al., 2006). Chymase, a heparin-bound serine protease with chymotrypsin-like activity, is one of the major components of cardiac mast cell granules. Mouse chymases include mouse mast cell protease (mMCP)-1, -4, -5, and -9. (Hellman and Thorpe, 2014). The closest mouse analog to human chymase, in terms of localization, substrate specificity and storage properties is mMCP-4 (Pejler et al., 2010). Furthermore, chymase activity in the mouse heart is primarily due to mMCP-4 expression (Houde et al., 2013). Cardiovascular targets of chymase in mice and humans include the activation of angiotensin-II (Ang-II), endothelin-1 (ET-1) and matrix metalloproteinase 9 (MMP-9) as well as the degradation of the matrix protein fibronectin (Balcells et al., 1997; Houde et al., 2013; Semaan et al., 2015; Caughey, 2016).

Chymase is rapidly secreted from mast cells after acute myocardial infarction (AMI) (Frangogiannis et al., 1998) and is upregulated in the infarcted region of human hearts (Daemen and Urata, 1997). Laine and colleagues also showed that the number of chymase-containing mast cells increases in the adventitia of coronary vessels of the infarcted region of human hearts (Laine et al., 1999). In heart failure models, such as permanent myocardial infarction (PMI) in the hamster, chymase inhibitors improve the cardiac function and survival, whether these molecules are used alone or in combination with an angiotensin-converting enzyme inhibitor (Hoshino et al., 2003; Jin et al., 2003). Chymase inhibition also reduced lethal arrhythmia 8 h after myocardial infarction in dogs, similarly to an angiotensin receptor antagonist, and reduces cardiomyofibrillar loss in a mitral regurgitation model of heart failure in this animal model (Jin et al., 2004; Pat et al., 2010). All these studies focused on the angiotensin-II (Ang-II)-producing activities of chymase, while Pat and colleagues further demonstrated chymase-dependent fibronectin degradation (Pat et al., 2010). Oyamada and colleagues also reported a reduction in active MMP-9 and infarcted area after chymase inhibition in a swine model of ischemia-reperfusion (Oyamada et al., 2011). On the other hand, Kanemitsu and colleagues also showed a beneficial action of chymase inhibition in a rat model of PMI, in which the action of chymase is thought to be Ang-II-independent, implying a transforming growth factor-β mechanism (Kanemitsu et al., 2006). In mouse settings, chymase inhibition reduces left ventricular remodeling in a model of heart failure caused by intermittent hypoxia (Matsumoto et al., 2009).

The expression of mMCP-4 is increased after PMI in mice (Ngkelo et al., 2016). Its genetic repression improves ventricular remodeling after ischemia-reperfusion injury (Tejada et al., 2016). This effect was shown to be independent from Ang-II system blockade and the authors suggested an insulin-like growth factor-1 (IGF-1), a previously unknown chymase substrate, dependent pathway (Tejada et al., 2016). ET-1 is also a major factor involved in myocardial infarction pathophysiology and is a critical factor in pulmonary hypertension, a possible complication of heart failure (Sakai et al., 1996). Finally our group demonstrated the role of mMCP-4 in the endogenous pulmonary synthesis of ET-1 *in vivo* (Houde et al., 2013). Therefore, in light of the beneficial role of chymase inhibitors in other rodent models of heart failure, we hereby hypothesized that specific mMCP-4 activity repression would protect mice from PMI. We demonstrate in the present study the profound impact of the genetic repression of mMCP-4 in mice subjected to irreversible left anterior descending (LAD) coronary artery ligation.

## Materials and Methods

Additional description is available in the **Supplementary Methods** section.

### Animals

C57BL/6J mice were purchased from Charles River (Montréal, QC, Canada) and housed in our facilities. Genitor mMCP-4 KO mice from the same background were generated by homologous recombination in embryonic stem cells as previously reported (Tchougounova et al., 2003) and bred in our facilities. Their homozygous genotypes were confirmed previously by our group (Houde et al., 2013). All animals were kept at constant room temperature (RT; 23°C) and humidity (78%) under a controlled light/dark cycle (6:00 AM–6:00 PM), with standard chow and tap water available *ad libitum*. Animal care and experiments were approved by the Ethics Committee on Animal Research of the University of Sherbrooke following the Canadian Council on Animal Care guidelines (Canadian Council on Animal Care [CCAC], 1993) and the Guide for the Care and Use of Laboratory Animals of the United States National Institutes of Health (National Research Council (US) Committee for the Update of the Guide for the Care and Use of Laboratory Animals, 2011).

### Animal Protocol

Four-month old male mice were anesthetized with a mixture of ketamine and xylazine (87/13 mg/kg, intra-muscular) and intubated to install assisted breathing. An incision was performed between the third and fourth ribs to access the LAD coronary artery. The pericardium was opened, and then PMI was induced when the LAD artery was ligated using 8-0 silk suture (Ethicon, Johnson & Johnson, Markham, ON, Canada) 2 mm from the atrium, and the costal cavity was then closed with 6-0 silk suture (Ethicon). SHAM surgery consisted in passing the suture under the LAD artery without ligating. Immediately after surgery, buprenorphine (0.1 mg/kg, subcutaneous) was administered for post-surgical pain management and subsequently added every 8 h for 24 h. The mice were distributed in four groups: wild-type (WT) SHAM, WT PMI, mMCP-4 KO SHAM, and mMCP-4 KO PMI. The mice were then monitored for 1 day (six mice per group) or 3 days (*n*: 15 WT SHAM, 12 mMCP-4 KO SHAM, 13 WT PMI, and 13 mMCP-4 KO PMI) or 7 days (*n*: 8 WT SHAM, 8 mMCP-4 KO SHAM, 8 WT PMI, and 8 mMCP-4 KO PMI). A subset (*n*: 9 WT SHAM, 6 mMCP-4 KO SHAM, 7 WT PMI, and 7 mMCP-4 KO PMI) of the 3-day protocol mice underwent positron emission tomography (PET) imaging as described below. The mice were then anesthetized and sacrificed and the hearts and lungs were collected, rinsed with isotonic NaCl (0.9%) to remove blood and put in formalin for histologic measurements or frozen at -80°C for protein analysis. Furthermore, another group of mice was monitored to establish a 4-week survival curve (7 SHAM and 15 PMI animals per genotype), and the surviving mice were sacrificed after completion of the 28-day protocol.

### Positron Emission Tomography

Seventy two hours after the induction of myocardial infarction, the mice were anesthetized with isoflurane and a catheter was inserted in the caudal vein to permit intra-venous administration of ^18^F-fluorodeoxyglucose (^18^F-FDG). The mice were then placed on the PET imaging bed with electrodes for electrocardiogram recording (ECG). A 45-min dynamic and synchronized data acquisition sequence in list mode was then launched simultaneously with the administration of ∼5 MBq of ^18^F-FDG in the caudal vein (100 μl infusion in 30 s). Glycaemia was measured before and again at the end of the imaging session, and another blood sample was collected, weighted and measured using a gamma radiation counter to normalize the blood time-activity curve. The mice were then euthanized by CO_2_ inhalation.

Reconstructed PET images were analyzed by tracing regions of interest (ROI) on the viable myocardium, left ventricular blood pool and liver to extract the tissue and blood time-activity curves. The blood input function was derived from the initial blood pool ROI and late liver ROI to overcome the spill-in problem of the left ventricular blood pool by the myocardium in the late portion of the time-activity curve. Pixel counts were corrected for radioactive decay of ^18^F and the counting efficiency of the PET scanner to obtain results in terms of percentage of injected dose per weight of tissue (%ID/g). We used the general solution of the three-compartment kinetic model of FDG to extract the myocardial metabolic rate of glucose (MMRG). Ventricular volumes and ejection fractions were calculated using standard clinical cardiac analysis software packages adapted to preclinical data. Polar maps were constructed by the reorientation of PET data to create a flat round representation of the myocardial external surface, with the apex in the middle as previously described (Kim et al., 2012). The infarcted area was then determined as the region with reduced tracer uptake.

### Microscopic Morphology Analysis

Formalin-fixed hearts were embedded in paraffin and 4 μm sections were cut in the short axis with a microtome. Selected sections were then deparaffinized in toluene, rehydrated in ethanol and water, then stained with hematoxylin and eosin (H&E), picrosirius red or toluidine blue (Moore and James, 1953). The slides were dehydrated with ethanol and then toluene, mounted with Permount medium (Fisher Scientific, Fair Lawn, NJ, United States) and imaged using a NanoZoomer 2.0-RS digital slide scanner (Hamamatsu Photonics, Hamamatsu, Japan).

Active (cleaved, Asp175) caspase-3 staining was performed using the rabbit anti-mouse active caspase-3 antibody (1:800, catalog # 9661, Cell Signaling Technology, ON, Canada) for a 30 min, RT incubation, after low pH antigen retrieval (EnVision FLEX Target Antigen Retrieval kit, Agilent, Montreal, QC, Canada). Detection was performed using horseradish peroxidase-linked goat anti-rabbit secondary antibody and revealed with the EnVision FLEX HRP high pH kit (Agilent) using the hydrogen peroxide and 3,3′-diaminobenzidine (DAB) chromophore system, with hematoxylin as counterstain.

Analysis of the images was performed with the NDP.view2 software (Hamamatsu Photonics), which provides direct absolute dimensions, such as area and length. The minimum ventricular free wall thickness was determined at the infarct site. Cross-sectional cardiomyocyte area was determined in the papillary muscle distal to the infarct site as previously described (Helms et al., 2010). Major and minor transverse axes and cross-sectional area were measured, and cardiomyocytes with an aspect ratio of ≥1.2 were excluded. This helped eliminate cells sectioned tangentially. Ten cardiomyocytes were measured per mouse heart section, with the average providing the value for the mouse specimen. Furthermore, inflammatory invasion and eosinophilia areas (both signs of cardiomyocyte death) were measured. The inflammatory invasion was defined as areas of nuclei-rich, eosin-poor staining, showing clearing of cardiomyocytes after necrosis and the invasion of multiple leukocytes. Eosinophilia is characterized by nuclei-free, more intensively eosin stained cardiomyocyte regions, showing cells in the process of necrosis. The total calculated area was corrected for the whole left ventricular wall with the NDP.view2 software. Image quantification was performed using ImageJ software.

### Proteomics

#### Sample Preparation for the LC-MS/MS Glycoproteomic Analysis

Left heart ventricles were reduced to a fine powder in liquid nitrogen using a mortar and a pestle. Soluble proteins were washed away by incubating the heart powder with PBS in the presence of protease inhibitors. Samples were centrifuged and the supernatant was discarded. Insoluble material was partly re-suspended in urea and subsequently re-centrifuged. The supernatant was removed and kept on ice (first solubilisation). The rest of the insoluble material was re-suspended in urea, thiourea and CHAPS, was centrifuged (second solubilisation) and the pellet was discarded. The soluble supernatants were then mixed together. Proteins were reduced with DTT and the total protein content was determined by colorimetry using Pierce 660 protein assay. Proteins were then alkylated with iodoacetamide in the dark, the reaction then quenched with additional DTT. Glycopeptides were oxidized with sodium periodate in the dark. Proteins were then precipitated by adding chilled acetone and the samples were placed at -80°C overnight. Precipitated proteins were aliquoted in several tubes and centrifuged. Pellets were washed twice with acetone:water (6:1) and then pooled and re-solubilized with urea-thiourea-CHAPS and in pH 5 sodium acetate. Glycoproteins were purified by adding hydrazide beads to the samples; unbound proteins were washed away with NaCl and then PBS. Proteins were digested with trypsin and then purified by reversed phase solid phase extraction, dried in a vacuum centrifuge, reconstituted in 0.2% formic acid and analyzed by LC-MS/MS.

#### LC-MS/MS Analysis

Acquisition was performed with a SCIEX TripleTOF 5600 (SCIEX, Foster City, CA, United States) equipped with an electrospray interface with a 25 μm iD capillary and coupled to an Eksigent μUHPLC (Eksigent, Redwood City, CA, United States). Analyst TF 1.6 software was used to control the instrument and for data processing and acquisition. Acquisition was performed in information dependant acquisition (IDA) mode for the protein database and in SWATH acquisition mode for the samples. For the SWATH acquisition, variable window sizes were used as computed by the SWATH Variable Window Calculator_V1.0 (SCIEX, Foster City, CA, United States). Separation was performed on a reversed phase HALO C18-ES column (Advance Materials Technology, Wilmington, DE, United States).

#### Data Analysis

Protein identification was performed with ProteinPilot V4.5 beta (SCIEX) with the instrument pre-set for TripleTOF5600. The ion libraries used for protein quantification were derived from the protein database generated by the IDA analysis of a pool of all the samples, which was injected twice in the MS. For protein quantification, data was analyzed using the PeakView software (SCIEX). A peptide was considered as adequately measured if the score computed by PeakView was superior to 0.5 or had a false discovery rate (FDR) < 1. Data is expressed as Log_2_ ratio of mMCP-4 KO/WT.

### Enzymatic Assays

Recombinant mMCP-4 (rmMCP-4) was produced in our facilities and activated with recombinant mouse cathepsin C (R&D Systems, Minneapolis, MN, United States) as previously described (Semaan et al., 2015). The rmMCP-4 (0.61 and 3.03 ng) was pre-incubated with either PBS or the chymase inhibitor TY-51469 (for a final concentration of 10 μM after substrate addition) at room temperature, then incubated with recombinant mouse IGF-1 (rmIGF-1, 1 μg, Sigma-Aldrich, St-Louis, MO, United States) or Big endothelin-1 (Big ET-1, 2.56 μg, Bachem, Torrance, CA, United States) in 21 μl for 20 min at 37°C. The reaction was stopped with an equal volume of acetonitrile 20% and formic acid 4% in water and the samples were frozen immediately.

Insulin-like growth factor-1 samples were then analyzed by LC-MS while Big ET-1 samples were analyzed by reverse-phase HPLC at 214 nm with a Zorbax DSC-18 column (Agilent Technologies, Montreal, QC, Canada).

### Tissue ET-1 Measurement

The lungs and left heart ventricle from WT and mMCP-4 KO mice were collected 1 and 3 days after surgery and frozen at -80°C until further use. On day of measurement, the samples were homogenized and purified as described before (Houde et al., 2013). Immunoreactive ET-1 (IR-ET-1) was then measured by ELISA (Quantikine ELISA, R&D Systems, Minneapolis, MN, United States) according to the manufacturer’s instructions.

### Plasma Brain Natriuretic Peptide (BNP) Measurement

Blood from WT and mMCP-4 KO mice were collected by right carotid artery catheterization 1 and 3 days after surgery in sodium citrate 3.5%, plasma was prepared by centrifugation at 1000 *g* for 15 min at RT; 4°C, after which the supernatant was collected and then frozen at -80°C until further use. On the day of measurement, the samples were thawed and directly assayed by ELISA for mouse BNP (MyBioSource, San Diego, CA, United States) according to the manufacturer’s recommendations.

### Statistics

Kaplan-Meier survival analysis was statistically tested with the Mantel-Cox method, PET and enzymatic assay statistical analyses were performed using a 1-way ANOVA followed by a Holms-Šidak post-test with Prism 7.0 software (GraphPad Software, La Jolla, CA, United States). One symbol (^∗^, #) denotes *P* < 0.05, two (^∗∗^, ##) show that *P* < 0.01, and three (^∗∗∗^, ###) show that *P* < 0.001. Glycoproteomic analysis was performed using Student’s *t*-test in Excel (Microsoft Corporation, Redmond, WA, United States) on data validated with the PeakView software.

## Results

### Mouse Survival

**FIGURE 1:**
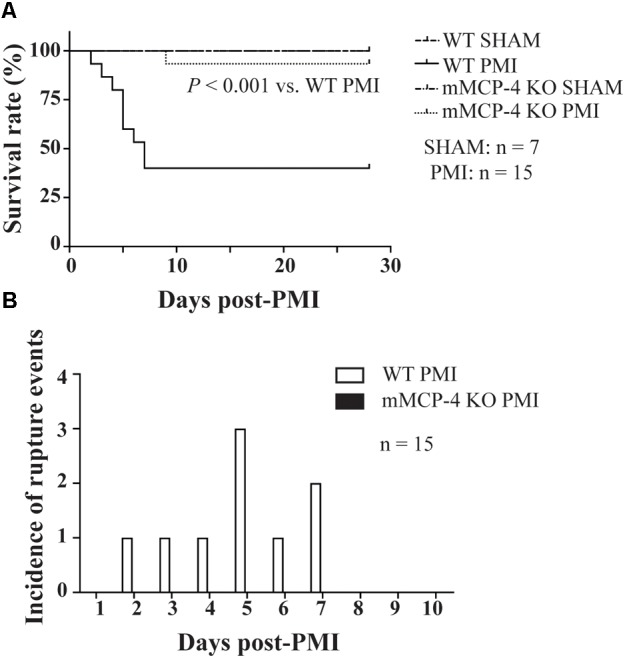
Kaplan-Meier 28-day survival analysis of mouse survival (Mantel-Cox test) **(A)** and incidence of left ventricular free wall rupture events **(B)** following LAD coronary artery ligation **(A)**. PMI: *n* = 15, SHAM: *n* = 7.

Permanent myocardial infarction led to 60% mortality in WT mice (**Figure 1A**). This mortality occurred in the first week following PMI, starting with one death at day two. In contrast, PMI only induced mortality in one mMCP-4 KO mouse at day nine, significantly less than in their WT congeners (*P* = 0.0014). SHAM surgery did not induce any mortality. Necropsy revealed that cardiac rupture was the cause of death in all 9 WT mice that did not complete the survival study, but no occurrence was found in mMCP-4 KO mice (**Figure 1B** and **Table 1**). Mouse heart weight, corrected to tibial bone length (which is independent of mouse fat content and variation due to the procedure), did not differ between genotypes or between mice that survived or died during the 28-day period (**Table 1**). A difference was observed, however, in mouse body weight change between surviving and dying WT mice, as surviving but not dying WT mice gained weight (*P* < 0.05). mMCP-4 KO mice that survived did not significantly lose weight. For subsequent experiments, PMI + 72 h was chosen to include all mice in our analyses before mortality events occurred.

**Table 1:** Mouse survival and weight changes after PMI.

Genotype	WT		mMCP-4 KO	
Status at 28 days	Alive	Dead	Alive	Dead
Number (rupture at autopsy)	6	9 (9)	14	1 (0)^∗^
Heart weight/tibial length (mg/mm)	10.39 ± 0.42	11.36 ± 0.42	10.04 ± 0.25	10.27
Body weight change (g)	1.93 ± 0.86	–3.54 ± 0.57^#^	–0.50 ± 0.72	–1.90

*The absolute value incidence of mortality and ventricular rupture for dead mice (in parentheses) is indicated, along with the heart weight (corrected with tibial length) and the total weight change after surgery. ^∗^ is vs. WT (rupture, Mantel-Cox test), ^#^ is vs. SHAM (one-way ANOVA with multiple comparisons Holms-Šidak post-test).*

### Cardiac Hemodynamics

**FIGURE 2:**
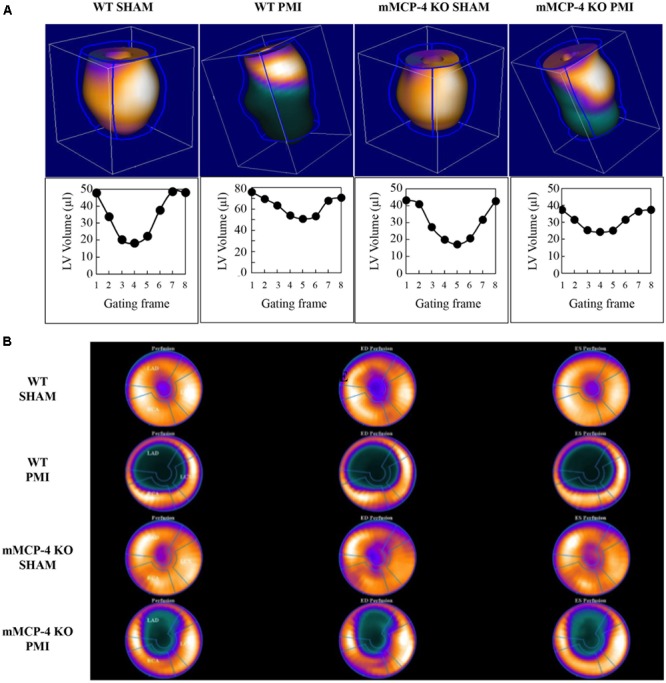
Representative cardiac PET imaging of mouse hearts after PMI **(A)**. Mouse hearts were imaged 3 days following surgery with ^18^F-FDG, and 3-dimension images were reconstructed. Below each reconstructed image is the ECG-gated left ventricular volume, measured through eight time points of the cardiac cycle (gating frames) for the same mouse. Polar maps of the mouse hearts were calculated, with the apex in the center, for the calculation of the infarcted area **(B)**.

Three days after ligation, the mice were subjected to ^18^F-FDG PET to evaluate cardiac function (representative images are shown in **Figure 2**). **Table 2** shows that PMI increased the left ventricular end-diastolic volume (LVEDV) in WT mice compared to SHAM. PMI also significantly increased the LVEDV in mMCP-4 KO mice. Moreover, the LVEDV remained significantly lower in infarcted mMCP-4 KO mice compared to their WT congeners, but this was not the case in SHAM animals. Similar changes were observed for the left ventricular end-systolic volume (LVESV), as PMI increased this parameter in WT and mMCP-4 KO, while it remained significantly lower in mMCP-4 KO mice compared to their WT congeners after PMI but not SHAM surgery.

**Table 2:** Left ventricular cardiac parameters and heart weight 3 days after surgery.

	WT		mMCP-4 KO	
	SHAM (9)	PMI (6)	SHAM (7)	PMI (7)
EDV (μl)	48.9 ± 5.4	76.7 ± 3.1^#^#	38.0 ± 3.8	60.3 ± 4.0^#^#,**
ESV (μl)	18.2 ± 3.2	49.5 ± 3.5^#^##	11.1 ± 3.0	32.9 ± 3.2^#^##,**
Ejection fraction (%)	65.1 ± 3.7	35.6 ± 2.1^#^##	73.6 ± 5.1	45.7 ± 3.6^#^##,*
Infarct size (%)	0	32.2 ± 2.6^#^##	0	24.1 ± 2.3^#^##,**
MMRG (μmol/100g/min)	112 ± 13	197 ± 17^#^#	125 ± 22	136 ± 8
Heart weight/tibial length (mg/mm)	8.23 ± 0.27	10.73 ± 0.62^#^##	7.63 ± 0.17	10.06 ± 0.20^#^##
Body weight change (g)	–1.86 ± 0.43	–5.17 ± 0.81^#^#	–1.73 ± 0.29	–3.51 ± 0.38^#^

*The left ventricular cardiac parameters were taken using ^*18*^F-FDG TEP, 3 days after SHAM surgery or the ligation of the LAD coronary artery, on isoflurane anesthetized mice. Sample size in parenthesis. # is vs. SHAM, ^∗^ is vs. WT (One-way ANOVA with multiple selected comparisons Holms-Šidak post-test). EDV, end-diastolic volume; ESV, end-systolic volume; MMRG, myocardial metabolic rate of glucose.*

Permanent myocardial infarction significantly reduced the ejection fraction (EF) in WT compared to SHAM operated mice. The mMCP-4 KO mice subjected to PMI exhibited a lower EF than their SHAM counterparts, but preserved their EF significantly better when compared to WT PMI controls.

### Cardiac Metabolism and Morphology

**FIGURE 3:**
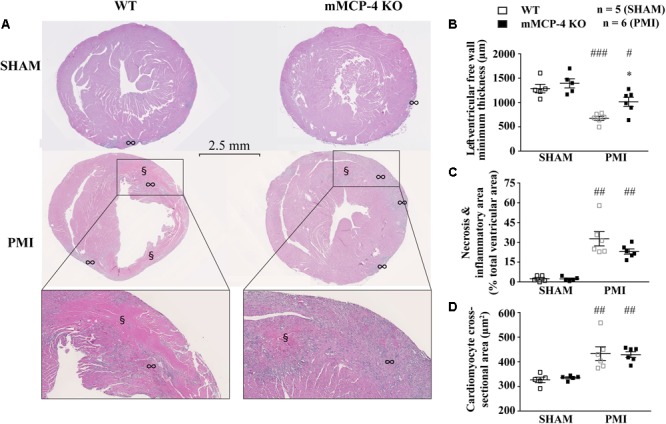
Representative H&E staining of heart sections 3 days following LAD coronary artery ligation **(A)**. § notes eosinophilia sites, ∞ marks inflammatory cell invasion sites. The minimal thickness at the infarct site **(B)**, eosinophilic and inflammatory regions **(C)** and cardiomyocyte cross-sectional area **(D)** were measured from the same images and quantified. (#: PMI vs. SHAM, ^∗^: mMCP-4 KO vs. WT, one-way ANOVA with multiple comparisons Holms-Šidak post-test).

Polar maps derived from ^18^F-FDG PET images (**Figure 2B**) were used to evaluate the infarcted area. SHAM surgery did not induce any visible infarction. The infarct area was significantly reduced in mMCP-4 KO compared to WT mice. H&E staining of formalin fixed heart slices showed extensive left ventricular remodeling in WT mice after PMI (**Figure 3** and **Supplementary Figure S1**). Toluidine blue staining revealed very few traces of mast cells in the myocardium, especially the infarcted area (**Supplementary Figure S1**), perhaps due to granule-poor mast cells after degranulation. It did, however, illustrate an increase in invading cells in the infarcted area of WT animals compared to mMCP-4 KO congeners 7 days post-PMI.

**FIGURE 4:**
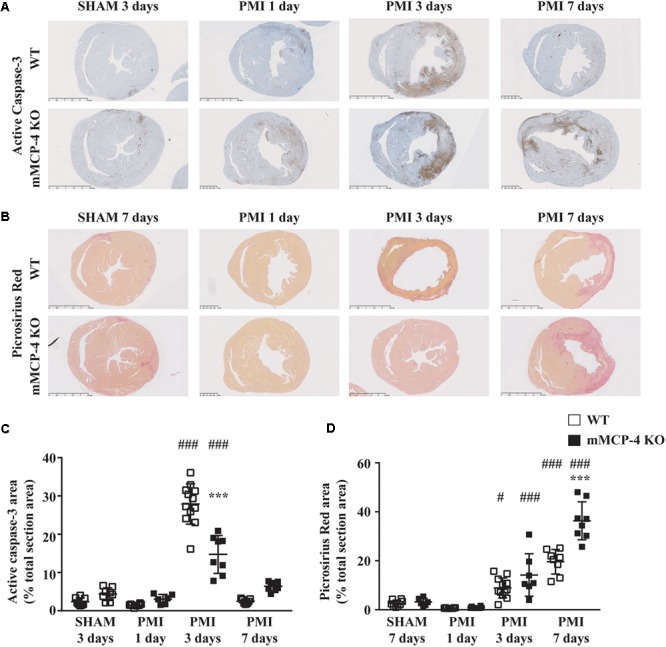
Representative active caspase-3 **(A)** and collagen deposition **(B)** of heart sections following LAD coronary artery ligation or SHAM surgery. Active caspase-3 DAB (brown) staining [quantified in **(C)**] is visible against the hematoxylin (blue) counterstain, while collagen deposition [quantified in **(D)**] is marked by the picrosirius red stain. (#: PMI vs. SHAM, ^∗^: mMCP-4 KO vs. WT, one-way ANOVA with multiple comparisons Holms-Šidak post-test).

Permanent myocardial infarction induced cardiomyocyte death (characterized by neutrophil invasion and eosinophilia of nuclei-free dying cells) in a similar fashion to the observed infarcted area as seen by ^18^F-FDG PET, although the difference between WT and mMCP-4 KO did not reach statistical significance in infarcted animals. Caspase-3 activation was increased after PMI, reaching a peak after 3 days and subsidizing by day seven (**Figure 4**). This increase was localized mainly in the infarcted area, and was more pronounced in WT mice at 3 days compared to their mMCP-4 KO congeners following PMI surgery (WT SHAM 3 days: 2.4 ± 0.4% of total section area, *n* = 8; WT PMI 1 day: 1.5 ± 0.1%, *n* = 8; WT PMI 3 days: 27.9 ± 1.5%, *n* = 12, *P* < 0.01 vs. WT SHAM 3 days; WT PMI 7 days: 2.5 ± 0.2%, *n* = 7; mMCP-4 KO SHAM 3 days: 4.3 ± 0.6%, *n* = 8; mMCP-4 KO PMI 1 day: 3.1 ± 0.5%, *n* = 6; mMCP-4 KO PMI 3 days: 14.7 ± 1.7%, *n* = 8, *P* < 0.001 vs. mMCP-4 KO SHAM 3 days, *P* < 0.001, vs. WT PMI 3 days, *P* < 0.001; and mMCP-4 KO PMI 7 days: 6.4 ± 0.4%, *n* = 8). Mice subjected to SHAM surgery showed signs of inflammation on the epicardium side of the left ventricular wall, but no cardiomyocyte necrosis inside the myocardium and no difference were noted between WT and mMCP-4 KO mice.

Three days after surgery, the minimum ventricular free wall thickness did not vary between WT and mMCP-4 KO mice subjected to SHAM surgery (WT SHAM: 1287 ± 88 μm and mMCP-4 KO SHAM: 1394 ± 94 μm, *n* = 5; **Figure 3**). PMI reduced the minimum free wall thickness after 3 days in both groups of mice compared to SHAM animals, but it remained significantly higher in mMCP-4 KO mice compared to their WT congeners (WT PMI: 676 ± 42 μm, *P* < 0.001 vs. WT SHAM; mMCP-4: 1015 ± 93 μm, *P* < 0.05 vs. mMCP-4 KO SHAM, *P* < 0.05 vs. WT PMI, *n* = 6). PMI also increased papillary cardiomyocyte cross-sectional area, but there was no difference between WT and mMCP-4 KO. Finally, picrosirius red staining (**Figure 4**) revealed that fibrosis in the infarcted area was already visible at 3 days in PMI mice, to reach a maximum at 7 days post-surgery (WT SHAM 7 days: 2.6 ± 0.5% of total section area, *n* = 8; WT PMI 1 day: 0.7 ± 0.04%, *n* = 8; WT PMI 3 days: 8.9 ± 1.2%, *n* = 12, *P* < 0.05 vs. WT SHAM 3 days; WT PMI 7 days: 19.6 ± 1.8%, *n* = 8; mMCP-4 KO SHAM 7 days: 3.2 ± 0.6%, *n* = 6; mMCP-4 KO PMI 1 day: 0.9 ± 0.1%, *n* = 8; mMCP-4 KO PMI 3 days: 14.2 ± 3.3%, *n* = 7, *P* < 0.001 vs. mMCP-4 KO SHAM 7 days; and mMCP-4 KO PMI 7 days: 36.3 ± 2.9%, *n* = 8). This increase in collagen deposition tended to be higher in mMCP-4 KO mice (*P* = 0.2) at 3 days post-PMI and reached statistical significance (*P* < 0.001) at 7 days post-PMI.

^18^F-fluorodeoxyglucose PET analysis also permits the monitoring of cardiac glucose consumption (**Table 2**). PMI increased MMRG in WT compared to mMCP-4 KO mice. Notably, the glucose consumption in infarcted mMCP-4 KO mouse hearts was similar to that of SHAM.

Heart hypertrophy was visible 3 days after PMI. Heart weight was elevated by PMI in WT compared to SHAM operated mice and mMCP-4 KO mice. There was no difference between WT and mMCP-4 KO mice after either SHAM or PMI surgery. WT mice also lost weight post-surgery, as did mMCP-4 KO mice.

**FIGURE 5:**
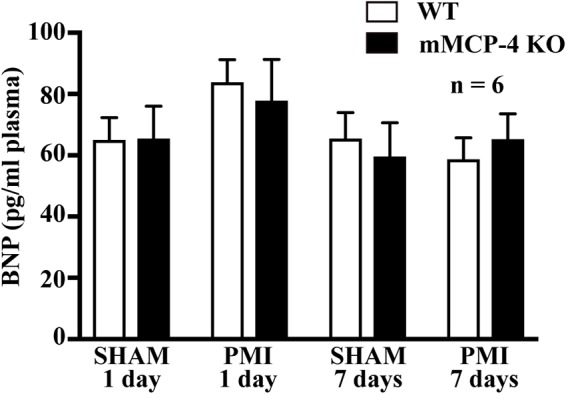
Plasma levels of BNP 1 or 7 days after SHAM or PMI surgery as measured by ELISA. *n* = 6 (one-way ANOVA *P* > 0.05).

One day post-PMI, plasma BNP levels were not different between WT and mMCP-4 KO mice after SHAM surgery (**Figure 5**) (WT SHAM: 65.0 ± 7.3 pg/ml, mMCP-4 KO SHAM: 65.4 ± 10.6 pg/ml, *n* = 6). PMI did not increase plasma BNP levels (WT PMI: 79.6 ± 7.0 pg/ml; mMCP-4 KO PMI: 70.9 ± 13.5 pg/ml, *n* = 6). Similar results were observed 72 h after surgery (WT SHAM: 65.48 ± 8.5 pg/ml; mMCP-4 KO SHAM: 59.6 ± 11.0 pg/ml, *n* = 6; WT PMI: 58.7 ± 7.0 pg/ml; and mMCP-4 KO PMI: 65.3 ± 8.2 pg/ml, *n* = 6). Plasma BNP levels from naïve animals were less than 50% of those from SHAM and PMI operated mice (data not shown), indicating that surgery (SHAM and PMI) increased plasma BNP levels similarly from baseline.

### Glycoproteomic Analysis

Glycoproteins were isolated from the left heart ventricles 72 h after PMI and were analyzed using TripleTOF mass spectrometry. A total of 999 glycoproteins were detected in SHAM and PMI mice (**Supplementary Table S1**). **Table 3** shows proteins whose levels were different between WT and mMCP-4 KO left ventricles only after PMI (but not in SHAM). Thirty seven glycoproteins were thus detected to be elevated (positive log_2_ ratio) or diminished (negative log_2_ ratio) in mMCP-4 KO mice compared to WT animals after PMI, with a cut-off of *P* < 0.05 using Student’s *t*-test (*n* = 6) for each protein. The single established chymase substrate for which a change was detected is highlighted in bold. Apolipoprotein A1 (ApoA1) was statistically increased in left ventricles of mMCP-4 KO vs. WT mice (*P* = 0.011). Most glycoproteins in the list are of mitochondrial origin. However, whereas certain mitochondrial proteins were upregulated due to the absence of chymase, others were downregulated. It is therefore not clear as to whether the absence of chymase leads to general effects on mitochondrial integrity. Two cardiac muscle proteins were found to be changed between WT and mMCP-4 KO mice following PMI: the upregulated tropomyosin β chain and the downregulated telethonin.

**Table 3:** Proteins differentially expressed (*P* < 0.05) in infarcted mMCP-4 KO mice compared to infarcted WT congeners.

Uniprot access code	Protein name	Log_2_ ratio
MYL1_MOUSE	Myosin light chain 1/3, skeletal muscle isoform (MLC1/MLC3)	7.56
B7ZCF1_MOUSE	26S protease regulatory subunit 6A	1.65
A2AIM4_MOUSE	Tropomyosin beta chain	1.39
G5E850_MOUSE	Cytochrome b-5, isoform CRA_a	1.32
**APOA1_MOUSE**	**Apolipoprotein A-I (Apo-AI)**	**0.76**
E9Q5L2_MOUSE	Inter alpha-trypsin inhibitor, heavy chain 4	0.69
ITIH3_MOUSE	Inter-alpha-trypsin inhibitor heavy chain H3	0.67
LMNA_MOUSE	Prelamin-A/C [cleaved into: lamin-A/C]	0.63
DHE3_MOUSE	Glutamate dehydrogenase 1, mitochondrial (GDH 1) (EC 1.4.1.3)	0.60
CES1D_MOUSE	Carboxylesterase 1D (carboxylesterase 3)	0.48
RL35_MOUSE	60S ribosomal protein L35	0.32
RL8_MOUSE	60S ribosomal protein L8	0.26
Q8C2Q8_MOUSE	ATP synthase subunit gamma	0.20
NDUB9_MOUSE	NADH dehydrogenase [ubiquinone] 1 beta subcomplex subunit 9 (complex I-B22)	–0.25
RSSA_MOUSE	40S ribosomal protein SA (37 kDa laminin receptor precursor)	–0.26
NDUA1_MOUSE	NADH dehydrogenase [ubiquinone] 1 alpha subcomplex subunit 1 (complex I-MWFE)	–0.27
KCRS_MOUSE	Creatine kinase *S*-type, mitochondrial (EC 2.7.3.2)	–0.28
TELT_MOUSE	Telethonin (titin cap protein)	–0.34
AT1B1_MOUSE	Sodium/potassium-transporting ATPase subunit beta-1	–0.36
RB11B_MOUSE	Ras-related protein Rab-11B	–0.36
NDUB6_MOUSE	NADH dehydrogenase [ubiquinone] 1 beta subcomplex subunit 6 (complex I-B17)	–0.37
PCCA_MOUSE	Propionyl-CoA carboxylase alpha chain, mitochondrial (PCCase subunit alpha)	–0.56
TBA4A_MOUSE	Tubulin alpha-4A chain (Alpha-tubulin 4)	–0.57
TIM50_MOUSE	Mitochondrial import inner membrane translocase subunit TIM50	–0.70
MOES_MOUSE	Moesin (membrane-organizing extension spike protein)	–0.90
PGK1_MOUSE	Phosphoglycerate kinase 1 (EC 2.7.2.3)	–0.93
RT4I1_MOUSE	Reticulon-4-interacting protein 1, mitochondrial	–1.09
GSTA4_MOUSE	Glutathione S-transferase A4 (EC 2.5.1.18) (GST A4-4)	–1.19
PLP2_MOUSE	Proteolipid protein 2	–1.56
G5E8T9_MOUSE	Hydroxyacyl glutathione hydrolase (mitochondrial)	–1.66
COQ3_MOUSE	Ubiquinone biosynthesis O-methyltransferase, mitochondrial (EC 2.1.1.114)	–1.69
DNPEP_MOUSE	Aspartyl aminopeptidase (EC 3.4.11.21)	–2.19
ATAD1_MOUSE	ATPase family AAA domain-containing protein 1 (EC 3.6.1.3) (thorase)	–2.30
PGAM1_MOUSE	Phosphoglycerate mutase 1	–2.73
EST1C_MOUSE	Carboxylesterase 1C (EC 3.1.1.1)	–2.78
E9Q7L0_MOUSE	Protein Ogdhl (oxyglutarate dehydrogenase-like protein)	–4.06
RMD1_MOUSE	Regulator of microtubule dynamics protein 1 (RMD-1)	–7.84

*A positive number signifies an increase in mMCP-4 KO mice, while a negative number means a decrease. A chymase substrate, Apolipoprotein A1, is highlighted in bold.*

### Tissue ET-1 Measurement

**FIGURE 6:**
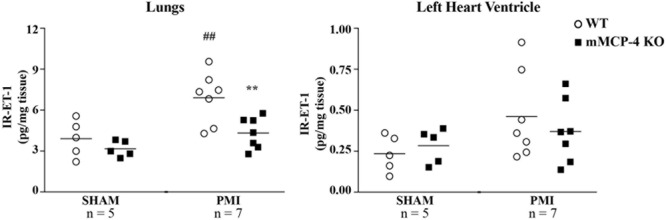
Tissue levels of IR-ET-1 1 day after surgery. The levels of immunoreactive ET-1 were measured in the lungs and left heart ventricle. #: PMI vs. SHAM, ^∗^: WT vs. mMCP-4 KO, *n* = 5–7, one-way ANOVA with multiple comparisons Holms-Šidak post-test).

One day post-PMI, tissue IR-ET-1 levels were not different between WT and mMCP-4 KO mice after SHAM surgery in either the left heart ventricle (WT SHAM: 0.056 ± 0.012 pg/mg, *n* = 5, mMCP-4 KO SHAM: 0.068 ± 0.011 pg/mg, *n* = 5) or lungs (WT SHAM: 5.21 ± 0.80 pg/mg, *n* = 5 and mMCP-4 KO SHAM: 4.22 ± 0.35 pg/mg, *n* = 5) (**Figure 6**). PMI showed a trend to increase left ventricular IR-ET-1 levels in WT mice, but this did not reach statistical significance (WT PMI: 0.111 ± 0.024 pg/mg, *n* = 7). PMI also did not increase IR-ET-1 levels in the mMCP-4 KO left heart ventricle, as it showed no statistically significant differences with SHAM or WT PMI (mMCP-4 KO PMI: 0.089 ± 0.017 pg/mg, *n* = 7). Similar results were obtained 3 days after surgery (WT PMI: 0.102 ± 0.021 pg/mg, *n* = 3; mMCP-4 KO PMI: 0.140 ± 0.022 pg/mg, *n* = 4). However, PMI caused a marked increase in pulmonary IR-ET-1 levels in the lungs of WT mice 1 day after surgery which was abolished in mMCP-4 KO mice (WT PMI: 9.21 ± 0.95 pg/mg, *n* = 7, *P* = 0.0052 vs. WT SHAM and mMCP-4 KO PMI: 5.77 ± 0.58 pg/mg, *n* = 7, *P* = 0.0065 vs. WT PMI). Pulmonary weight did not vary following PMI surgery (WT SHAM 1 day: 5.5 ± 0.1 mg/g body weight, *n* = 7, WT PMI 1 day: 5.5 ± 0.1 mg/g, *n* = 10, WT PMI 3 days: 6.3 ± 0.4 mg/g, *n* = 10, WT PMI 7 days: 6.8 ± 0.7 mg/g, *n* = 11; mMCP-4 KO SHAM: 5.4 ± 0.1 mg/g, *n* = 10; mMCP-4 KO PMI 1 day: 5.8 ± 0.3 mg/g, *n* = 9, mMCP-4 KO PMI 3 days: 6.2 ± 0.2 mg/g, *n* = 10 and mMCP-4 KO PMI 7 days: 6.7 ± 0.3 mg/g, *n* = 10).

### Enzymatic Assays

**FIGURE 7:**
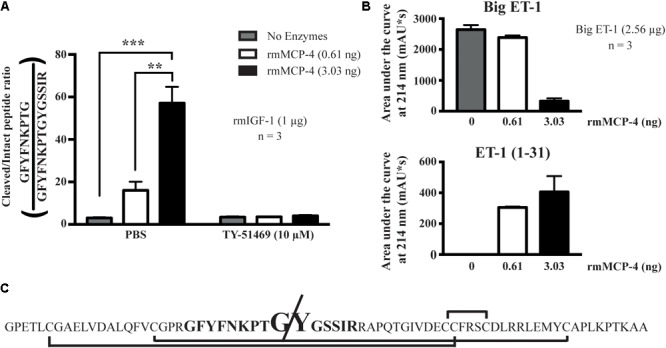
Analysis of the *in vitro* hydrolysis of recombinant mouse IGF-1 (LC/MS/MS) and Big ET-1 (HPLC) by recombinant mMCP-4, with the chymase inhibitor TY-51469 (10 μM). In **(A)**, the results of recombinant mMCP-4 activity on mouse IGF-1 are shown using trypsin-degradation products, with GFYFNKPTGYGSSIR coming from the complete IGF-1 sequence and GFYFNKPTGY coming from the mMCP-4-cleaved sequence. In **(B)**, the area under the curve (AUC) was measured for Big ET-1 and ET-1 (1–31) using HPLC at 214 nm detection. *n* = 3 (one-way ANOVA with multiple comparisons Holms-Šidak post-test). In **(C)**, the complete sequence of mouse IGF-1 is shown, with the disulfide bonds shown with brackets, the analyzed trypsin-digested product in bold and the cleavage site in increased type size.

Recombinant mMCP-4 showed rmIGF-1 cleaving activity *in vitro* (**Figure 7A**). At 0.61 ng, a quantity previously shown by our group to convert Big ET-1 to ET-1 (1–31) efficiently (Semaan et al., 2015), rmMCP-4 did not significantly produce the cleaved rmIGF-1 fragment (**Figure 7A**) although it processed Big ET-1 to ET-1 (1–31) (**Figure 7C**). However, increasing the amount of rmMCP-4 to 3.03 ng led to a significant production of the cleaved IGF-1 peptide but led to an almost complete degradation of Big ET-1 without further increasing detected ET-1 (1–31) levels compared to 0.61 ng. Treatment with TY-51469 (10 μM) completely blocked rmIGF-1 degradation. We also determined that chymase cleaves the Tyr^31^-Gly^32^ bond of mouse IGF-1 (**Figure 7B**).

## Discussion

Our study is the first to report the impact of the genetic repression of mMCP-4 in the context of myocardial infarction after permanent LAD coronary artery ligation. This mouse isoform is the closest to human chymase in terms of tissue localization, storage properties and substrate specificity. Thus, mMCP-4 represents a relevant model for the study of the pathophysiological role of chymase in human disease (Pejler et al., 2010). Our results show that PMI induced a 60% mortality rate in WT mice due to left ventricular free wall thinning and rupture. The wall thinning was reduced in mMCP-4 KO mice, which showed no sign of wall rupture and mortality was almost entirely prevented in the later murine strain. We also show that the repression of mMCP-4 improves cardiac parameters compared to WT mice after PMI, in addition to reducing the infarcted zone, apoptosis signaling and improving energy metabolism. Glycoproteomic analysis of the changes in glycoprotein levels between WT and mMCP-4 KO mice after PMI revealed that ApoA1 was elevated in the left ventricles of mMCP-4 KO mice. Finally, one day after PMI, the levels of ET-1 in the lungs, but not the heart, were elevated in WT but not mMCP-4 KO mice.

Pharmacological inhibition of chymase improves survival in the hamster, rat, and dog after the induction of PMI (Jin et al., 2002, 2003, 2004; Kanemitsu et al., 2006). These inhibitors have, however, not been tested for selectivity against mMCP-4 nor against another mouse β-chymase with angiotensinase activity such as mMCP-1. We have previously shown that mMCP-4 deletion reduces chymase-like activity in mouse hearts, while mMCP-1 mRNA levels were unchanged (Houde et al., 2013). Thus, the present results suggest for the first time that chymase activity inhibition improves survival in mice following PMI due to impairment of mMCP-4 activity.

Ventricular rupture is a major factor involved in mouse mortality following permanent ligation of the LAD coronary artery (Unsold et al., 2014; Sutton et al., 2017). The left ventricular free wall was thinner in WT than mMCP-4 KO mice following PMI, perhaps leading to the observed ventricular wall rupture in those animals. As mMCP-4 can activate numerous matrix degrading enzymes (such as gelatinases) and directly degrades matrix proteins like fibronectin (Tchougounova et al., 2003, 2005), the absence of mMCP-4 may protect the ventricular wall from degradation and rupture. One possible mechanism illustrated in our study is through the increased collagen deposition in the free left ventricular wall. This result is of interest since chymase, through its activation of factors, such as Ang-II, MMP-9, and TGF-β, is reputed to be pro-fibrotic (Levick and Widiapradja, 2018). Although cardiac fibrosis is detrimental to long-term heart function, initial matrix deposition is crucial to the initiation of myocardial repair (Frangogiannis, 2014). The direct effect of chymase on myocardial fibroblasts is less clear, as results from Carver and colleagues suggest that chymase reduces cardiac fibroblast production of extracellular matrix and their conversion to the myofibroblast phenotype, while Hooshdaran and collaborators found that chymase and cathepsin G promote differentiation into myofibroblasts, although they did not differentiate between these two close proteases (Carver et al., 2015; Hooshdaran et al., 2017). Chymase, together with its activation of MMP-9, is also a direct matrix degrading enzyme and may reduce fibroblast pro-collagen-1 production (Fu et al., 2016). Therefore, removing chymase from mast cells might protect fibroblast function and the overall pro-fibrotic signals of mast cells (Ngkelo et al., 2016), preventing matrix degradation to the point of rupture. Furthermore, chymase promotes apoptosis of smooth muscle cells (den Dekker et al., 2012), and may do so in cardiomyocyte as well (Hooshdaran et al., 2017). Our results show an increase in active caspase-3 staining in WT mice compared to mMCP-4 KO animals, indicating an increase in apoptosis signaling in cardiomyocytes, albeit our results cannot distinguish cell types outside of the eosinophilic, cardiomyocyte-specific regions. Taken together, these results suggest a protective role for chymase inhibition by reducing ventricular free wall thinning through matrix degradation and apoptosis after PMI.

^18^F-fluorodeoxyglucose PET cardiac analysis has been previously shown in the mouse model to reliably estimate cardiac parameters (Stegger et al., 2009). To study cardiac function in PMI mice, we chose the 72 h post-PMI mark to include as many mice as possible and to represent the peak of chymase activity following PMI in hamsters (Jin et al., 2001). Albeit PMI significantly altered ventricular dilation and ejection fraction in both WT and mMCP-4 KO mice, these changes were less pronounced in the chymase-deficient animals. Similar results were observed with the pharmacological inhibition of chymase in hamsters 3 days post-PMI (Jin et al., 2003). Ngkelo and colleagues in contrast, suggested no difference in left ventricular function 14 days after permanent ligation of the LAD coronary artery in mMCP-4 KO mice albeit no supporting data was provided (Ngkelo et al., 2016). Thus, chymase activity inhibition appears to protect cardiac function immediately following PMI in rodents, perhaps by limiting remodeling as reflected by our H&E staining. This is in contrast to the ischemia-reperfusion model, where mMCP-4 deletion improves cardiac function no earlier than 14 days post-surgery, reflecting the different pathological development of heart failure in this model (van Zuylen et al., 2015; Tejada et al., 2016). Clearly, there is discrepancy in the data regarding the effect of chymase inhibition on the depression of left ventricular hemodynamic parameters following myocardial infarction.

Compared to other common cardiac imaging techniques such as echocardiography and magnetic resonance imaging, ^18^F-FDG PET loses resolution for hemodynamic and morphologic analyses, but permits infarct size evaluation *in vivo* (Stegger et al., 2006) as well as glucose consumption measurement (Lecomte et al., 2004; Mabrouk et al., 2012). The balance between lipid and glucose consumption by the heart for its energy requirements is shifted from a mostly lipidic to glycemic regime in the infarcted and remodeled regions after myocardial infarction, as glucose consumption is more oxygen-efficient than fatty acid metabolism in the hypoxic tissue (Stanley et al., 2005). While our imaging study did not consider fatty acid uptake by the mouse heart, glucose consumption was elevated in WT, but not mMCP-4 KO hearts following PMI. This may reflect a greater distress state in WT hearts, as the switch from fatty acid to glucose metabolism may not occur in the very early stages of heart failure and occur later in mMCP-4 KO mice (Stanley et al., 2005). This is the first study suggesting that chymase inhibition improves the metabolic state of the failing heart, but complementary data such as lipidic metabolism and later time points are needed in future experiments to confirm this activity.

We observed a decreased infarcted zone after PMI in mMCP-4 KO mice compared to their WT congeners. This is in contrast with studies in which pharmacological chymase inhibitors have been used in the hamster after PMI (Jin et al., 2002, 2003; Hoshino et al., 2003). It should be noted that those reports used stacked histochemistry analysis to estimate infarct size. In our study, the decreased infarcted size in mMCP-4 KO did not reach statistical significance in our 2D H&E analysis, while the 3D analysis by ^18^F-FDG PET revealed a similar, but statistically significant, reduction. Thus, methodological differences could explain this discrepancy between our study and the previous reports with chymase inhibitors. Furthermore, chymase inhibition or deletion is able to reduce the infarct size in other models of heart failure, such as ischemia-reperfusion in the swine (Oyamada et al., 2011) or the mouse (Tejada et al., 2016); this may be due to reduced inflammation-induced cardiomyocyte death when chymase activity is suppressed. The observed beneficiary effects of mMCP-4 deletion on left ventricular dilation and ejection fraction, though statistically significant only in mice subjected to PMI, are similar to those on SHAM animals. Thus, protection of ventricular wall integrity may provide the main mechanism for the promotion of survival by mMCP-4 deletion following PMI, as it is also observed in mice deficient for the ectonucleotidase CD39 (Sutton et al., 2017) or overexpressing the molecular chaperon melusin (Unsold et al., 2014). Furthermore, we did not observe significant fibrosis in either WT or mMCP-4 KO mice, suggesting that differences in fibrotic mechanisms do not account in the differences in survival between mouse genotypes.

Few studies measured plasma BNP after myocardial infarction in mice, revealing widely variating results. For instance, Petrera and colleagues measured less than 15 pg/ml in infarcted mice, while Thireau and co-workers measured more than 5 ng/ml in the same model and the same ELISA kit provider (Thireau et al., 2012; Petrera et al., 2016). Those studies showed an increase of plasma BNP compared to SHAM animals 4 weeks after the induction of myocardial infarction, but their SHAM surgeries did not involve passing the suture under the LAD coronary artery without ligating. Interestingly, in our study, plasma BNP levels were elevated to the same levels by SHAM and PMI surgery, indicating that, in contrast to clinical settings, this peptide is not a useful biomarker for myocardial infarction severity in mice. It does indicate that our SHAM animals did suffer from a minor infarction, as seen with the histological staining, but without any impact on cardiac function or viability.

In the current glycoproteomic study, well-established chymase substrates went undetected (such as MMP-9) and left ventricular fibronectin (**Supplementary Table S1**) levels did not vary between WT and mMCP-4 KO PMI mice. However, an interesting new identified candidate for cardioprotection in PMI is the chymase substrate ApoA1 (Kokkonen et al., 1986). Chymase inhibits ApoA1 cholesterol efflux and anti-inflammatory activity through C-terminal cleavage (Lindstedt et al., 1996; Nguyen et al., 2016), which may explain our results even in normolipidemic settings. Furthermore, HDL levels are inversely correlated with percutaneous coronary intervention-related myocardial infarction risks (Sattler et al., 2009) and ApoA1 plasma levels correlate positively with improved outcomes in coronary artery bypass graft surgery patients, suggesting a protective role in ischemic heart disease (Skinner et al., 1999). Activation of the receptor for sphingosine-1-phosphate, a HDL component, also reduces infarct size in a pig ischemia-reperfusion model (Santos-Gallego et al., 2014, 2016). Very recently, Kareinen et al. (2018) demonstrated, in a Langendorff-perfused rat heart system, that low flow ischemia induced mast cell degranulation and chymase-like degradation of ApoA1, suggesting a deficiency in endothelial healing processes following myocardial injury. Taken together, our results combined with the literature suggest that chymase inhibition-mediated protection of HDL integrity could explain the lower infarct size after myocardial infarction. Chymase inhibition also protects against cardiomyofibrillar loss in a dog model of mitral regurgitation (Pat et al., 2010), a process which may be reflected by the measured increased of tropomyosin β-chain and our histochemical results.

Endothelin-1 is an early protective factor in heart failure *in vivo* and the immediate antagonism of its receptors reduces survival and worsens remodeling in the rat after PMI (Nguyen et al., 1998, 2001). However, ET-1 is a potent mediator of congestive heart failure-induced pulmonary hypertension (Sakai et al., 1996), yet pharmacological blockade of endothelin receptors does not improve its parameters in animal models. This suggests that inhibition of ET-1 synthesis by chymase blockade may provide a better pharmacological approach to prevent this complication of PMI (Nguyen et al., 2000; Jiang et al., 2011), although we did not find evidence of pulmonary inflammation or fibrosis in our model. Since it has no effect on left ventricular levels of ET-1, either after surgery (present study) or endogenously (Houde et al., 2013), inhibition of pulmonary located chymase may provide the desired repression of the ET-1 system without impeding its beneficial cardiac effect after PMI. Tejada and colleagues suggested a detrimental role of mMCP-4 in the degradation of IGF-1 during ischemia-reperfusion injury in the mouse (Tejada et al., 2016). IGF-1, like Big ET-1, possesses potential chymotrypsin-sensitive sites (Takaoka et al., 1990; Anderle et al., 2002). We found that mMCP-4 cleaves the Tyr^31^-Gly^32^ bond of IGF-1, but very high concentrations of the recombinant enzyme are required to hydrolyze this peptide. Although our results do not rule out a role of mMCP-4 dependent degradation of IGF-1 in our model, we consider unlikely that direct degradation of the growth factor by chymase is responsible for this action *in vivo*.

Ventricular rupture could account to up to 20% of all myocardial infarction related deaths in humans. Reperfusion is the best clinical prevention of this complication, explaining why there are no rupture events in ischemia-reperfusion experimental models where the induced ischemia is comparatively short (Figueras et al., 2000). Thus, there is a need for more severe disease models such as the permanent ligation of the LAD coronary artery, which is etiologically different from the human clinical situation but more reflective of its outcomes such as ventricular rupture (Gao et al., 2005). Our study is the first to report the effect of genetic chymase activity repression on ventricular rupture, joining other recently reported strategies effective in the mouse model (Unsold et al., 2014; Sutton et al., 2017). Thus, future studies should establish the impact of pharmacological chymase inhibition on rupture events in the mouse PMI model. The mechanism for cardiac protection due to chymase inhibition also needs to be refined. For example, it would be interesting to determine the role of ApoA1 degradation by chymase, in the context of myocardial infarction.

## Conclusion

In conclusion, our results show that chymase repression improves survival and cardiac parameters in a mouse model of PMI. If the present study in the mouse model can be extended to the clinical settings in human subjects, our study suggests that chymase inhibition may be beneficial in the treatment of myocardial infarction.

## Author Contributions

MH participated in the study design, experimental work, data analysis and interpretation throughout the study, and authored the first draft of the article and was responsible for managing its editing. AS participated in the study design, data analysis and interpretation, as well as the editing of the manuscript. HT participated in the *in vivo* experimental work, histological stainings, BNP and ET-1 measurements, and contributed to the manuscript. LD participated in the *in vivo* experimental work and the editing of the manuscript. OS contributed to the *in vivo* imaging modalities and data analysis. RL contributed to the *in vivo* imaging modalities of the study, participated in the design of that experiment and contributed to the manuscript writing and editing. ML contributed to the *in vivo* imaging modalities of the study and contributed to the manuscript writing and editing. HG contributed the mass spectrometry experimental work of the study, including sample preparation, participated in data analysis, and contributed to the writing. ST contributed to the writing and editing of the manuscript. GP contributed the mouse model and to the editing of the manuscript. DJ participated in data analysis for histological experiments, and contributed to the manuscript editing. FG contributed to the active caspase-3 staining and the editing of the manuscript. RD contributed to the editing of the manuscript and in the production of recombinant mMCP-4. PD-J initiated and participated in the study design, data analysis and interpretation, article writing and editing, supervised and provided the funding for the study.

## Conflict of Interest Statement

HG is employed by PhenoSwitch Bioscience Inc. (Sherbrooke, Canada). The remaining authors declare that the research was conducted in the absence of any commercial or financial relationships that could be construed as a potential conflict of interest.

## Abbreviations

^18^F-FDG: ^18^F-fluorodeoxyglucose
AMI: acute myocardial infarction
ET-1: endothelin-1
IGF-1: insulin-like growth factor-1
LAD: left anterior descending coronary artery
mMCP-4: mouse mast cell protease-4
PET: positron emission tomography
PMI: permanent myocardial infarction

## References

[B1] Anderle P. Langguth P. Rubas W. Merkle H. P. (2002). In vitro assessment of intestinal IGF-I stability. *J. Pharm. Sci.* 91 290–300. 10.1002/jps.10013 11782919

[B2] Balcells E. Meng Q. C. Johnson W. H. Jr. Oparil S. Dell’Italia L. J. (1997). Angiotensin II formation from ACE and chymase in human and animal hearts: methods and species considerations. *Am. J. Physiol.* 273(4 Pt 2) H1769–H1774. 10.1152/ajpheart.1997.273.4.H1769 9362242

[B3] Canadian Council on Animal Care [CCAC] (1993). *Guide for the Care and Use of Experimental Animals.* Ottawa: Canadian Council on Animal Care.

[B4] Carver W. Carver A. Fix C. Goldsmith E. (2015). Effects of mast cell chymase on cardiac fibroblast function. *FASEB J.* 29(Suppl. 1) 972–974. 10.1096/fasebj.29.1_supplement.972.4

[B5] Caughey G. H. (2016). Mast cell proteases as pharmacological targets. *Eur. J. Pharmacol.* 778 44–55. 10.1016/j.ejphar.2015.04.045 25958181 PMC4636979

[B6] Daemen M. J. Urata H. (1997). Healing human myocardial infarction associated with increased chymase immunoreactivity. *Heart Vessels Suppl.* 12 113–115. 9476559

[B7] den Dekker W. K. Tempel D. Bot I. Biessen E. A. Joosten L. A. Netea M. G. (2012). Mast cells induce vascular smooth muscle cell apoptosis via a toll-like receptor 4 activation pathway. *Arterioscler. Thromb. Vasc. Biol.* 32 1960–1969. 10.1161/ATVBAHA.112.250605 22652603

[B8] Figueras J. Cortadellas J. Soler-Soler J. (2000). Left ventricular free wall rupture: clinical presentation and management. *Heart* 83 499–504. 10.1136/heart.83.5.499 10768896 PMC1760810

[B9] Frangogiannis N. G. (2014). The inflammatory response in myocardial injury, repair, and remodelling. *Nat. Rev. Cardiol.* 11 255–265. 10.1038/nrcardio.2014.28 24663091 PMC4407144

[B10] Frangogiannis N. G. Lindsey M. L. Michael L. H. Youker K. A. Bressler R. B. Mendoza L. H. (1998). Resident cardiac mast cells degranulate and release preformed TNF-alpha, initiating the cytokine cascade in experimental canine myocardial ischemia/reperfusion. *Circulation* 98 699–710. 10.1161/01.CIR.98.7.699 9715863

[B11] Fu L. Wei C. C. Powell P. C. Bradley W. E. Ahmad S. Ferrario C. M. (2016). Increased fibroblast chymase production mediates procollagen autophagic digestion in volume overload. *J. Mol. Cell Cardiol.* 92 1–9. 10.1016/j.yjmcc.2016.01.019 26807691 PMC5198899

[B12] Gao X. M. Xu Q. Kiriazis H. Dart A. M. Du X. J. (2005). Mouse model of post-infarct ventricular rupture: time course, strain- and gender-dependency, tensile strength, and histopathology. *Cardiovasc. Res.* 65 469–477. 10.1016/j.cardiores.2004.10.014 15639486

[B13] Hellman L. Thorpe M. (2014). Granule proteases of hematopoietic cells, a family of versatile inflammatory mediators - an update on their cleavage specificity, in vivo substrates, and evolution. *Biol. Chem.* 395 15–49. 10.1515/hsz-2013-0211 23969467

[B14] Helms S. A. Azhar G. Zuo C. Theus S. A. Bartke A. Wei J. Y. (2010). Smaller cardiac cell size and reduced extra-cellular collagen might be beneficial for hearts of Ames dwarf mice. *Int. J. Biol. Sci.* 6 475–490. 10.7150/ijbs.6.475 20827400 PMC2935670

[B15] Hooshdaran B. Kolpakov M. A. Guo X. Miller S. A. Wang T. Tilley D. G. (2017). Dual inhibition of cathepsin G and chymase reduces myocyte death and improves cardiac remodeling after myocardial ischemia reperfusion injury. *Basic Res. Cardiol.* 112:62. 10.1007/s00395-017-0652-z 28913553 PMC6287604

[B16] Hoshino F. Urata H. Inoue Y. Saito Y. Yahiro E. Ideishi M. (2003). Chymase inhibitor improves survival in hamsters with myocardial infarction. *J. Cardiovasc. Pharmacol.* 41(Suppl. 1) S11–S18. 12688390

[B17] Houde M. (2017). *Implication de la Protéase Mastocytaire 4 dans la Biosynthèse de L’endothéline-1 le Développement de L’athérosclérose et L’infarctus du Myocarde.* Ph.D. thesis, Sherbrooke, QC, Université de Sherbrooke.

[B18] Houde M. Jamain M. D. Labonte J. Desbiens L. Pejler G. Gurish M. (2013). Pivotal role of mouse mast cell protease 4 in the conversion and pressor properties of Big-endothelin-1. *J. Pharmacol. Exp. Ther.* 346 31–37. 10.1124/jpet.112.202275 23596057 PMC3684843

[B19] Jiang B. H. Tardif J. C. Shi Y. Dupuis J. (2011). Bosentan does not improve pulmonary hypertension and lung remodeling in heart failure. *Eur. Respir. J.* 37 578–586. 10.1183/09031936.00053710 20595149

[B20] Jin D. Takai S. Sakaguchi M. Okamoto Y. Muramatsu M. Miyazaki M. (2004). An antiarrhythmic effect of a chymase inhibitor after myocardial infarction. *J. Pharmacol. Exp. Ther.* 309 490–497. 10.1124/jpet.103.061465 14730006

[B21] Jin D. Takai S. Yamada M. Sakaguchi M. Kamoshita K. Ishida K. (2003). Impact of chymase inhibitor on cardiac function and survival after myocardial infarction. *Cardiovasc. Res.* 60 413–420. 10.1016/S0008-6363(03)00535-2 14613871

[B22] Jin D. Takai S. Yamada M. Sakaguchi M. Miyazaki M. (2002). Beneficial effects of cardiac chymase inhibition during the acute phase of myocardial infarction. *Life Sci.* 71 437–446. 10.1016/S0024-3205(02)01689-2 12044843

[B23] Jin D. Takai S. Yamada M. Sakaguchi M. Yao Y. Miyazaki M. (2001). Possible roles of cardiac chymase after myocardial infarction in hamster hearts. *Jpn. J. Pharmacol.* 86 203–214. 10.1254/jjp.86.203 11459123

[B24] Kanemitsu H. Takai S. Tsuneyoshi H. Nishina T. Yoshikawa K. Miyazaki M. (2006). Chymase inhibition prevents cardiac fibrosis and dysfunction after myocardial infarction in rats. *Hypertens. Res.* 29 57–64. 10.1291/hypres.29.57 16715654

[B25] Kareinen I. Baumann M. Nguyen S. D. Maaninka K. Anisimov A. Tozuka M. (2018). Chymase released from hypoxia-activated cardiac mast cells cleaves human apolipoproteinA-I at Tyr192 and compromises its cardioprotective activity. *J. Lipid Res.* 56:jlr.M077503. 10.1194/jlr.M077503 29581158 PMC5983404

[B26] Kim D. Y. Kim H. S. Le U. N. Jiang S. N. Kim H. J. Lee K. C. (2012). Evaluation of a mitochondrial voltage sensor, (18F-fluoropentyl)triphenylphosphonium cation, in a rat myocardial infarction model. *J. Nucl. Med.* 53 1779–1785. 10.2967/jnumed.111.102657 23038748

[B27] Kokkonen J. O. Vartiainen M. Kovanen P. T. (1986). Low density lipoprotein degradation by secretory granules of rat mast cells. Sequential degradation of apolipoprotein B by granule chymase and carboxypeptidase A. *J. Biol. Chem.* 261 16067–16072. 3536921

[B28] Laine P. Kaartinen M. Penttila A. Panula P. Paavonen T. Kovanen P. T. (1999). Association between myocardial infarction and the mast cells in the adventitia of the infarct-related coronary artery. *Circulation* 99 361–369. 10.1161/01.CIR.99.3.361 9918522

[B29] Lecomte R. Croteau E. Gauthier M.-E. Archambault M. Aliaga A. Rousseau J. (2004). Cardiac PET imaging of blood flow, metabolism and function in normal and infarcted rats. *IEEE Trans. Nucl. Sci.* 51 696–704. 10.1109/TNS.2004.829608

[B30] Levick S. P. Melendez G. C. Plante E. McLarty J. L. Brower G. L. Janicki J. S. (2011). Cardiac mast cells: the centrepiece in adverse myocardial remodelling. *Cardiovasc. Res.* 89 12–19. 10.1093/cvr/cvq272 20736239 PMC3002871

[B31] Levick S. P. Widiapradja A. (2018). Mast cells: key contributors to cardiac fibrosis. *Int. J. Mol. Sci.* 19:E231. 10.3390/ijms19010231 29329223 PMC5796179

[B32] Lindstedt L. Lee M. Castro G. R. Fruchart J. C. Kovanen P. T. (1996). Chymase in exocytosed rat mast cell granules effectively proteolyzes apolipoprotein AI-containing lipoproteins, so reducing the cholesterol efflux-inducing ability of serum and aortic intimal fluid. *J. Clin. Invest.* 97 2174–2182. 10.1172/JCI118658 8636396 PMC507296

[B33] Mabrouk R. Dubeau F. Bentourkia M. Bentabet L. (2012). Extraction of time activity curves from gated FDG-PET images for small animals’ heart studies. *Comput. Med. Imaging Graph* 36 484–491. 10.1016/j.compmedimag.2012.05.002 22658459

[B34] Matsumoto C. Hayashi T. Kitada K. Yamashita C. Miyamura M. Mori T. (2009). Chymase plays an important role in left ventricular remodeling induced by intermittent hypoxia in mice. *Hypertension* 54 164–171. 10.1161/HYPERTENSIONAHA.109.131391 19470876

[B35] Moore J. E. James G. W. (1953). A simple direct method for absolute basophil leukocyte count. *Proc. Soc. Exp. Biol. Med.* 82 601–603. 10.3181/00379727-82-20190 13055944

[B36] National Research Council (US) Committee for the Update of the Guide for the Care and Use of Laboratory Animals (2011). *Guide for the Care and Use of Laboratory Animals.* Washington, DC: National Academies Press. 21595115

[B37] Ngkelo A. Richart A. Kirk J. A. Bonnin P. Vilar J. Lemitre M. (2016). Mast cells regulate myofilament calcium sensitization and heart function after myocardial infarction. *J. Exp. Med.* 213 1353–1374. 10.1084/jem.20160081 27353089 PMC4925026

[B38] Nguyen Q. T. Cernacek P. Calderoni A. Stewart D. J. Picard P. Sirois P. (1998). Endothelin A receptor blockade causes adverse left ventricular remodeling but improves pulmonary artery pressure after infarction in the rat. *Circulation* 98 2323–2330. 10.1161/01.CIR.98.21.2323 9826321

[B39] Nguyen Q. T. Cernacek P. Sirois M. G. Calderone A. Lapointe N. Stewart D. J. (2001). Long-term effects of nonselective endothelin A and B receptor antagonism in postinfarction rat: importance of timing. *Circulation* 104 2075–2081. 10.1161/hc4201.097187 11673349

[B40] Nguyen Q. T. Colombo F. Rouleau J. L. Dupuis J. Calderone A. (2000). LU135252, an endothelin(A) receptor antagonist did not prevent pulmonary vascular remodelling or lung fibrosis in a rat model of myocardial infarction. *Br. J. Pharmacol.* 130 1525–1530. 10.1038/sj.bjp.0703466 10928953 PMC1572223

[B41] Nguyen S. D. Maaninka K. Lappalainen J. Nurmi K. Metso J. Oorni K. (2016). Carboxyl-terminal cleavage of apolipoprotein A-I by human mast cell chymase impairs its anti-inflammatory properties. *Arterioscler. Thromb. Vasc. Biol.* 36 274–284. 10.1161/ATVBAHA.115.306827 26681753 PMC4725095

[B42] Oyamada S. Bianchi C. Takai S. Chu L. M. Sellke F. W. (2011). Chymase inhibition reduces infarction and matrix metalloproteinase-9 activation and attenuates inflammation and fibrosis after acute myocardial ischemia/reperfusion. *J. Pharmacol. Exp. Ther.* 339 143–151. 10.1124/jpet.111.179697 21795433 PMC11047277

[B43] Pat B. Chen Y. Killingsworth C. Gladden J. D. Shi K. Zheng J. (2010). Chymase inhibition prevents fibronectin and myofibrillar loss and improves cardiomyocyte function and LV torsion angle in dogs with isolated mitral regurgitation. *Circulation* 122 1488–1495. 10.1161/CIRCULATIONAHA.109.921619 20876440 PMC3092363

[B44] Pejler G. Ronnberg E. Waern I. Wernersson S. (2010). Mast cell proteases: multifaceted regulators of inflammatory disease. *Blood* 115 4981–4990. 10.1182/blood-2010-01-257287 20233968

[B45] Petrera A. Gassenhuber J. Ruf S. Gunasekaran D. Esser J. Shahinian J. H. (2016). Cathepsin A inhibition attenuates myocardial infarction-induced heart failure on the functional and proteomic levels. *J. Transl. Med.* 14:153. 10.1186/s12967-016-0907-8 27246731 PMC4888645

[B46] Sakai S. Miyauchi T. Kobayashi M. Yamaguchi I. Goto K. Sugishita Y. (1996). Inhibition of myocardial endothelin pathway improves long-term survival in heart failure. *Nature* 384 353–355. 10.1038/384353a0 8934519

[B47] Santos-Gallego C. G. Badimon J. J. Rosenson R. S. (2014). Beginning to understand high-density lipoproteins. *Endocrinol. Metab. Clin. North Am.* 43 913–947. 10.1016/j.ecl.2014.08.001 25432389

[B48] Santos-Gallego C. G. Vahl T. P. Goliasch G. Picatoste B. Arias T. Ishikawa K. (2016). Sphingosine-1-phosphate receptor agonist fingolimod increases myocardial salvage and decreases adverse postinfarction left ventricular remodeling in a porcine model of ischemia/reperfusion. *Circulation* 133 954–966. 10.1161/CIRCULATIONAHA.115.012427 26826180

[B49] Sattler K. J. Herrmann J. Yun S. Lehmann N. Wang Z. Heusch G. (2009). High high-density lipoprotein-cholesterol reduces risk and extent of percutaneous coronary intervention-related myocardial infarction and improves long-term outcome in patients undergoing elective percutaneous coronary intervention. *Eur. Heart J.* 30 1894–1902. 10.1093/eurheartj/ehp183 19474052

[B50] Semaan W. Desbiens L. Houde M. Labonte J. Gagnon H. Yamamoto D. (2015). Chymase inhibitor-sensitive synthesis of endothelin-1 (1-31) by recombinant mouse mast cell protease 4 and human chymase. *Biochem. Pharmacol.* 94 91–100. 10.1016/j.bcp.2015.02.001 25667044

[B51] Skinner J. S. Farrer M. Albers C. J. Neil H. A. Adams P. C. (1999). High apolipoprotein AI concentrations are associated with lower mortality and myocardial infarction five years after coronary artery bypass graft surgery. *Heart* 81 488–494. 10.1136/hrt.81.5.488 10212166 PMC1729029

[B52] Stanley W. C. Recchia F. A. Lopaschuk G. D. (2005). Myocardial substrate metabolism in the normal and failing heart. *Physiol. Rev.* 85 1093–1129. 10.1152/physrev.00006.2004 15987803

[B53] Stegger L. Heijman E. Schafers K. P. Nicolay K. Schafers M. A. Strijkers G. J. (2009). Quantification of left ventricular volumes and ejection fraction in mice using PET, compared with MRI. *J. Nucl. Med.* 50 132–138. 10.2967/jnumed.108.056051 19091898

[B54] Stegger L. Hoffmeier A. N. Schafers K. P. Hermann S. Schober O. Schafers M. A. (2006). Accurate noninvasive measurement of infarct size in mice with high-resolution PET. *J. Nucl. Med.* 47 1837–1844. 17079817

[B55] Sutton N. R. Hayasaki T. Hyman M. C. Anyanwu A. C. Liao H. Petrovic-Djergovic D. (2017). Ectonucleotidase CD39-driven control of postinfarction myocardial repair and rupture. *JCI Insight* 2:e89504. 10.1172/jci.insight.89504 28097233 PMC5213916

[B56] Takaoka M. Miyata Y. Takenobu Y. Ikegawa R. Matsumura Y. Morimoto S. (1990). Mode of cleavage of pig big endothelin-1 by chymotrypsin. Production and degradation of mature endothelin-1. *Biochem. J.* 270 541–544. 10.1042/bj2700541 2205205 PMC1131757

[B57] Tchougounova E. Lundequist A. Fajardo I. Winberg J. O. Abrink M. Pejler G. (2005). A key role for mast cell chymase in the activation of pro-matrix metalloprotease-9 and pro-matrix metalloprotease-2. *J. Biol. Chem.* 280 9291–9296. 10.1074/jbc.M410396200 15615702

[B58] Tchougounova E. Pejler G. Abrink M. (2003). The chymase, mouse mast cell protease 4, constitutes the major chymotrypsin-like activity in peritoneum and ear tissue. A role for mouse mast cell protease 4 in thrombin regulation and fibronectin turnover. *J. Exp. Med.* 198 423–431. 10.1084/jem.20030671 12900518 PMC2194091

[B59] Tejada T. Tan L. Torres R. A. Calvert J. W. Lambert J. P. Zaidi M. (2016). IGF-1 degradation by mouse mast cell protease 4 promotes cell death and adverse cardiac remodeling days after a myocardial infarction. *Proc. Natl. Acad. Sci. U.S.A.* 113 6949–6954. 10.1073/pnas.1603127113 27274047 PMC4922143

[B60] Thireau J. Karam S. Fauconnier J. Roberge S. Cassan C. Cazorla O. (2012). Functional evidence for an active role of B-type natriuretic peptide in cardiac remodelling and pro-arrhythmogenicity. *Cardiovasc. Res.* 95 59–68. 10.1093/cvr/cvs167 22617407

[B61] Unsold B. Kaul A. Sbroggio M. Schubert C. Regitz-Zagrosek V. Brancaccio M. (2014). Melusin protects from cardiac rupture and improves functional remodelling after myocardial infarction. *Cardiovasc. Res.* 101 97–107. 10.1093/cvr/cvt235 24130190

[B62] van Zuylen V. L. den Haan M. C. Roelofs H. Fibbe W. E. Schalij M. J. Atsma D. E. (2015). Myocardial infarction models in NOD/Scid mice for cell therapy research: permanent ischemia vs ischemia-reperfusion. *Springerplus* 4:336. 10.1186/s40064-015-1128-y 26185738 PMC4498004

[B63] Zhang Q. Y. Ge J. B. Chen J. Z. Zhu J. H. Zhang L. H. Lau C. P. (2006). Mast cell contributes to cardiomyocyte apoptosis after coronary microembolization. *J. Histochem. Cytochem.* 54 515–523. 10.1369/jhc.5A6804.2005 16344327

