# Supplementary material for: Mouse Mast Cell Protease 4 Deletion Protects Heart Function and Survival After Permanent Myocardial Infarction
Source: Front Pharmacol. 2018 Aug 31;9:868. doi: 10.3389/fphar.2018.00868 (PMC6127244; doi:10.3389/fphar.2018.00868)
Supplement: Supplementary file 2 [file Data_Sheet_1.pdf]

Supplementary Material

MOUSE MAST CELL PROTEASE 4 DELETION PROTECTS HEART FUNCTION AND SURVIVAL AFTER ACUTE MYOCARDIAL INFARCTION

Martin Houde[1,2], Adel Schwertani[3], Hanène Touil[1], Louisane Desbiens[1], Otman Sarrhini[4], Roger Lecomte[4], Martin Lepage[4], Hugo Gagnon[5], Shinji Takai[6], Gunnar Pejler[7,8], Danielle Jacques[9], Robert Day[10], Fernand Gobeil[1], Pedro D'Orléans-Juste[1*]

Correspondence: Pedro D'Orléans-Juste,

labpdj@usherbrooke.ca

Supplementary Methods

Positron Emission Tomography

PET imaging experiments and data analysis were performed at the Sherbrooke Molecular Imaging Centre of CRCHUS using mostly methods previously described (Croteau et al., 2003; Menard et al., 2009). 72 h after the induction of myocardial infarction, the mice were anesthetised with isoflurane (1-2 % inhalation in 1.0-1.5 L/min pure oxygen with spontaneous respiration) and a drop of blood was collected from a hind leg to measure glycaemia. A catheter was then inserted in the caudal vein to permit the intra-venous administration of ^{18}F-fluorodeoxyglucose (^{18}F-FDG). The anesthetised mice were then placed on the bed of a LabPET-4 small animal PET scanner (Advanced Molecular Imaging (AMI) Inc., Sherbrooke, QC, Canada) having a 3.75 cm axial field of view. Electrodes were placed for recording the electrocardiogram (ECG) using a PCSAM model 1025T synchronisation system (SA Instruments Inc., Stony Brook, NY, USA). The mouse was then positioned for its heart to be in the center of the scanner view field. Simultaneously with the injection of ~5 MBq of FDG in the caudal vein (100 µl infusion in 30 sec), a list mode PET data acquisition sequence was initiated and continued for 45 min, while recording ECG and monitoring

the animal hemodynamic parameters (heart and breathing rates, O_2 saturation). Body temperature was controlled through forced warm air. At the end of the imaging session, glycaemia was measured again in a drop of blood from a hind leg. Another blood sample was collected, weighted and measured using a gamma radiation well-counter to determine the blood radioactivity concentration. This sample was used to normalise the blood and tissue time-activity curves in units of Bq/ml. The mice were then euthanized by CO_2 inhalation, and the heart was collected and frozen at -80 °C for further analyses.

List-mode data were first divided in 43 frames according to the sequence 1 × 30 s, 16 × 5 s, 7 × 10 s, 8 × 30 s, 1 × 60 s, 5 × 150 s and 5 × 300 s for the kinetic analysis of the ^{18}F-FDG uptake rate. PET image frames (120 × 120 × 128 pixels, 0.5 x 0.5 x 0.59675 mm^3/pixel) were then reconstructed with the MLEM (maximum likelihood expectation maximisation) iterative method implementing the physical description of the system matrix. The last 15 min of the acquisition sequence were summed and used to manually draw regions of interest (ROI) on the viable myocardium, the left ventricular blood pool and the liver. The same data were used to obtain an 8-gate image series of the cardiac cycle using the ECG timestamps recorded in the list mode file.

The tissue uptake curves and blood input functions were obtained by copying ROIs on the dynamic image series and measuring the mean ROI count rates per frame. The method developed by Tantawy and Petersen (Tantawy and Peterson, 2010) was used to avoid the problem associated with the contamination of the left ventricular blood pool by the myocardium in estimating the blood input function. Briefly, the blood radioactivity concentration as a function of time is a hybrid curve consisting of the first 2 min of the left ventricular blood pool, followed by the 2-45 min of the liver time-activity curve. Pixel counts were corrected for the radioactive decay of ^{18}F, referred

to the time of injection, and the counting efficiency of the PET scanner to obtain results in terms of percentage of injected dose per weight of tissue (%ID/g).

To estimate the glucose consumption, the general solution of the classical 3-compartment, 5-parameter kinetic model of ^{18}F-FDG (Phelps et al., 1979), where the myocardial metabolic rate of glucose or MMRG is in units of µmol/100 g/min. Ventricular volumes, ejection fractions and polar maps were calculated with the Corridor4DM-preclinical software (INVIA Medical Imaging Solutions, Ann Harbor, MI, USA).

Proteomics

Sample preparation for the LC-MS/MS glycoproteomic analysis

Hearts were reduced to a fine powder in liquid nitrogen using a mortar and a pestle. Soluble proteins were washed away by incubating the heart powder in 1 ml of phosphate buffer saline (PBS) + 0.5 M NaCl + 1 X protease inhibitors for 30 min at 4 °C with end over end mixing. Samples were centrifuged at 13 000 RPM for 5 min, at 4 °C and the supernatant was discarded. Insoluble material was partly resuspended in 1 ml of 8 M urea, 0.4 M NH_4HCO_3 and 0.1 % sodium deoxycholate for 10 min at room temperature (RT) with occasional vortexing. Samples were then centrifuged at 13 000 RPM for 5 min at RT. The supernatant was removed and kept on ice (first solubilisation). The rest of the insoluble material was resuspended in 7 M urea + 2 M thiourea + 4 % CHAPS in sodium acetate pH 5, incubated at RT for another 10 min with occasional vortex and pipette ups and downs. Samples were clarified by centrifugation at 13 000 RPM for 5 min at RT (second solubilisation) and the pellet was discarded. The supernatant was added to the supernatant of the first solubilisation and mixed by vortexing. Proteins were reduced by adding 10 mM of DTT at 60 °C for 15 min and the total protein content was determined by colorimetry using Pierce 660

protein assay. Proteins were then alkylated by adding 15 mM iodoacetamide for 30 min at RT, in the dark. The reaction was quenched with the addition of 10 mM DTT. Glycopeptides were oxidized with 10 mM sodium periodate for 30 min at RT in the dark. Proteins were transferred to a 15 ml tube and precipitated by adding one volume (2 ml) of chilled acetone and vortexing briefly. 5 more volumes (10 ml) of chilled acetone were added to the samples, the tubes were vortexed briefly and placed at -80 °C overnight. Precipitated proteins were placed in several 2 ml Eppendorf tubes and centrifugated for 1 min at 13 000 RPM. Protein pellets were washed twice with 2 ml acetone:water (6:1). Pellets of the same condition were pooled and resolubilized in 500 µl of 7 M urea + 2 M thiourea + 4 % CHAPS in sodium acetate pH 5 with pipette ups and downs. Glycoproteins were purified by adding 50 µl of hydrazide beads in 500 µl of 0.1 M sodium acetate pH 5 to the samples and mixing end over end overnight at RT. Unbound proteins were washed away with 3 successive 5 min incubation with 1.5 M NaCl. Beads and bound glycoproteins were washed 3 more times in phosphate buffer pH 7.5. Proteins were digested in 100 µl of phosphate buffer + 0.75 M urea + 2 µg trypsin for 4 h at 42 °C and the peptides were purified by solid phase extraction (SPE). SPE was performed with a polymeric mixed mode strong cation exchange 1 ml cartridge containing 30 mg of sorbent (Phenomenex, Strata-X-C, Torrance, CA, USA). Briefly samples were pre-treated with 2 % FA and loaded on the SPE column, samples were next washed once with 60 % MeOH containing 2 % formic acid (FA) and eluted with 2 x 700 µl of 75 % acetonitrile (CAN) containing 10 % ammonium hydroxide. The peptides were finally dried in a speedvac, reconstituted in 20 µl of water + 0.2 % FA and analyzed by LC-MS/MS.

LC-MS/MS analysis

Acquisition was performed with a Sciex TripleTOF 5600 (Sciex, Foster City, CA, USA) equipped with an electrospray interface with a 25 µm iD capillary and coupled to an Eksigent

µUHPLC (Eksigent, Redwood City, CA, USA). Analyst TF 1.6 software was used to control the instrument and for data processing and acquisition. The source voltage was set to 5.2 kV and maintained at 225 °C, curtain gas was set at 27 psi, gas one at 12 psi and gas two at 10 psi. Acquisition was performed in Information Dependant Acquisition (IDA) mode for the protein database and in SWATH acquisition mode for the samples. For the SWATH acquisition, variable window sizes were used as computed by the SWATH Variable Window Calculator_V1.0 (Sciex, Foster City, CA, USA) Separation was performed on a reversed phase HALO C18-ES column 0.3 µm i.d., 2.7 µm particles, 150 mm long (Advance Materials Technology, Wilmington, DE) which was maintained at 60 °C. Samples were injected by loop overfilling into a 5 µl loop. For the 75 min LC gradient, the mobile phase consisted of the following solvent A (0.2 % v/v FA and 3 % dimethyl sulfoxide (DMSO) v/v in water) and solvent B (0.2 % v/v FA and 3 % DMSO in EtOH) at a flow rate of 3 µl/min. The gradient was: 0-44 min from 2 % to 40 % B, 44-54 min from 40 % to 65 % B, 54-57 min from 65 % to 95 % B, hold 95 % B from 57 to 75 min, followed by a 5 min post-flush at final conditions.

Data analysis

Protein identification was performed with ProteinPilot V4.5 beta (Sciex) with the instrument pre-set for TripleTOF5600, iodoacetamide as cys alkylation as special factor. Thorough search with false discovery rate analysis was performed with biological modification emphasis against the mouse proteome. For protein identification and data analysis global false discovery rate was set at 1 % and local false discovery rate (FDR) was set at 5 %. The ion libraries used for protein quantification were derived from the protein database generated by the IDA analysis of a pool of all the samples, which was injected twice in the MS. For protein quantification, data was analyzed using the Peakview software (Sciex) using 8 transition/peptide and 15 peptide/protein

maximum. A peptide was considered as adequately measured if the score computed by Peakview was superior to 0.5 or had a FDR < 1.

Enzymatic Assays

Sample preparation

Samples were reduced with 10 mM DTT at 65 °C for 15 minutes in 50 mM Tris pH 8.0 and alkylated with 15 mM iodoacetamide at room temperature in the dark for 30 minutes. This reaction was then quenched with an additional 10 mM DTT. Samples were diluted in 1 ml of 50 mM Tris pH 8.0 + 0.1 % sodium deoxycholate and 0.25 µg of Trypsin/LysC was added. The digestion was carried over night at 37 °C with agitation. Samples were acidified with 2 % FA and purified by reversed phase SPE as described above.

LC-MS analysis

Acquisition was performed in positive mode with a TripleTOF 5600 (Sciex, Foster City, CA, USA) equipped with an electrospray interface with a 50 µm iD capillary and coupled to an Eksigent µUHPLC (Eksigent, Redwood City, CA, USA). Analyst TF 1.7 software was used to control the instrument and for data processing and acquisition. The source voltage was set to 5.1 kV and maintained at 225 °C, curtain gas was set at 27 psi, gas one at 12 psi and gas two at 2 psi. Acquisition was performed in MRM mode. The detailed transition, declustering potential and collision energy are reported in the table S1. Separation was performed on a reversed phase HALO C18 column 0.5 µm i.d., 2.7 µm particles, 50 mm long (Advance Materials Technology, Wilmington, DE) which was maintained at 50 °C. Samples were injected by loop overfilling into a 5 µL loop. For the 2.5 min LC gradient, the mobile phase consisted of the following solvent A (0.2 % v/v FA v/v in water) and solvent B (0.2% v/v FA in ACN) at a flow rate of 25 µL/min.

Peptide quantification

Peptides were quantified with the MultiQuant software (Sciex, Foster City, CA, USA). Peak integration was manually inspected for each sample, as well as for each transition. The quantification of a peptide represents the sum of the area under the curve (AUC) from several transitions (see Table S1 for details).

Table S1: Detection details for peptide quantification

**Peptide sequence**	**Q1 (m/z)**	**MS/MS transition (m/z)**	**DP (V)**	**CE (V)**
GFYFNKPTGY	597.3	826.41<br>679.34<br>437.2	28.267	100
GFYFNKPTGYGSSIR (2+)	847.4	989.47<br>847.44<br>937.46	40.52	100
GFYFNKPTGYGSSIR (3+)	565.3	937.46<br>745.37	25.13	100

Enzymatic assays

Recombinant mMCP-4 (rmMCP-4) was produced in our facilities and activated with recombinant murine cathepsin C (R&D Systems, Minneapolis, MN, USA) as previously described (Semaan et al., 2015). Recombinant mMCP-4 was thawed and diluted to a concentration of 20 µg/ml in maturation buffer (50 mM MES, 0.1 % BSA, pH 5.5). Active murine cathepsin C was

diluted to 0.481 ng/ml in cathepsin C buffer (50 mM MES, 50 mM NaCl, 5 mM DTT, pH 5.5). Activation was performed by adding equal volumes of rmMCP-4 and cathepsin C, adding 50 µg/ml heparin and incubating 1 hour at RT. Chymase activation was stopped with N-ethylmaleimide (NEM) (3 mM) and diluted with assay buffer (20 mM Tris, 2M KCl, 0.02 % Triton X-100, pH 9.0) to bring the recombinant chymase concentration to 2 µg/ml, and 5 min was afforded to completely stop the cathepsin C-dependent reaction. The rmMCP-4 (0.61 and 3.03 ng) was pre-incubated with either PBS or the chymase inhibitor TY-51469 (for a final concentration of 10 µM after substrate addition) at room temperature, then incubated with recombinant mouse insulin growth factor-1 (rm-IGF-1, 1µg) or Big endothelin-1 (Big ET-1, 2.56 µg) in 21 µl for 20 minutes at 37 °C. The reaction was stopped with an equal volume of ACN 20 % and FA 4% in water and the samples were frozen immediately.

IGF-1 samples were then thawed and the buffer was neutralised. The samples were reduced with DTT, alkylated with iodoacetamide and then digested by trypsin overnight and purified by reverse-phase solid phase extraction as described above. LC-MS analysis was performed in positive mode with a TripleTOF 5600 system (Sciex, Foster City, CA, USA) and the results were quantified with the MultiQuant software (Sciex). The quantification represents the sum of the area under the curve (AUC) from several transitions.

Big ET-1 conversion to ET-1 (1-31) was measured by reverse-phase HPLC (Agilent Technologies, Montreal, QC, CAN) using a Zorbax DSC-18 column. Detection was performed with 214 nm ultraviolet light, and quantification was performed with the AUC.

Tissue ET-1 measurement

The lungs and left ventricle from WT and mMCP-4 KO mice were collected 1 and 3 days after surgery and frozen at -80 °C until further use. On day of measurement, the samples were homogenised in a chloroform-methanol solution (1:4) using a glass homogeniser on ice, water was added and the samples were centrifuged at 2500 *g* at 4 °C for 15 min. The aqueous phase was then collected and acidified with one volume of trifluoroacetic acid (TFA) 0.2%. The samples were then purified on a DSC-18 solid phase extraction column (Supelco, Bellefonte, PA, USA) and eluted with 60% ACN-0.1% TFA. The resulting samples were then dried overnight and the IR-ET-1 was measured by ELISA (Quantikine ELISA, R&D Systems, Minneapolis, MN) according to the manufacturer's instructions.

Supplementary References

Croteau, E., Benard, F., Cadorette, J., Gauthier, M.E., Aliaga, A., Bentourkia, M., et al. (2003). Quantitative gated PET for the assessment of left ventricular function in small animals. *J Nucl Med* 44(10), 1655-1661.

Menard, S.L., Ci, X., Frisch, F., Normand-Lauziere, F., Cadorette, J., Ouellet, R., et al. (2009). Mechanism of reduced myocardial glucose utilization during acute hypertriglyceridemia in rats. *Mol Imaging Biol* 11(1), 6-14. doi: 10.1007/s11307-008-0171-2.

Phelps, M.E., Huang, S.C., Hoffman, E.J., Selin, C., Sokoloff, L., and Kuhl, D.E. (1979). Tomographic measurement of local cerebral glucose metabolic rate in humans with (F-18)2-fluoro-2-deoxy-D-glucose: validation of method. *Ann Neurol* 6(5), 371-388. doi: 10.1002/ana.410060502.

Semaan, W., Desbiens, L., Houde, M., Labonte, J., Gagnon, H., Yamamoto, D., et al. (2015). Chymase inhibitor-sensitive synthesis of endothelin-1 (1-31) by recombinant mouse mast cell protease 4 and human chymase. *Biochem Pharmacol* 94(2), 91-100. doi: 10.1016/j.bcp.2015.02.001.

Tantawy, M.N., and Peterson, T.E. (2010). Simplified [18F]FDG image-derived input function using the left ventricle, liver, and one venous blood sample. *Mol Imaging* 9(2), 76-86.

Supplementary Results

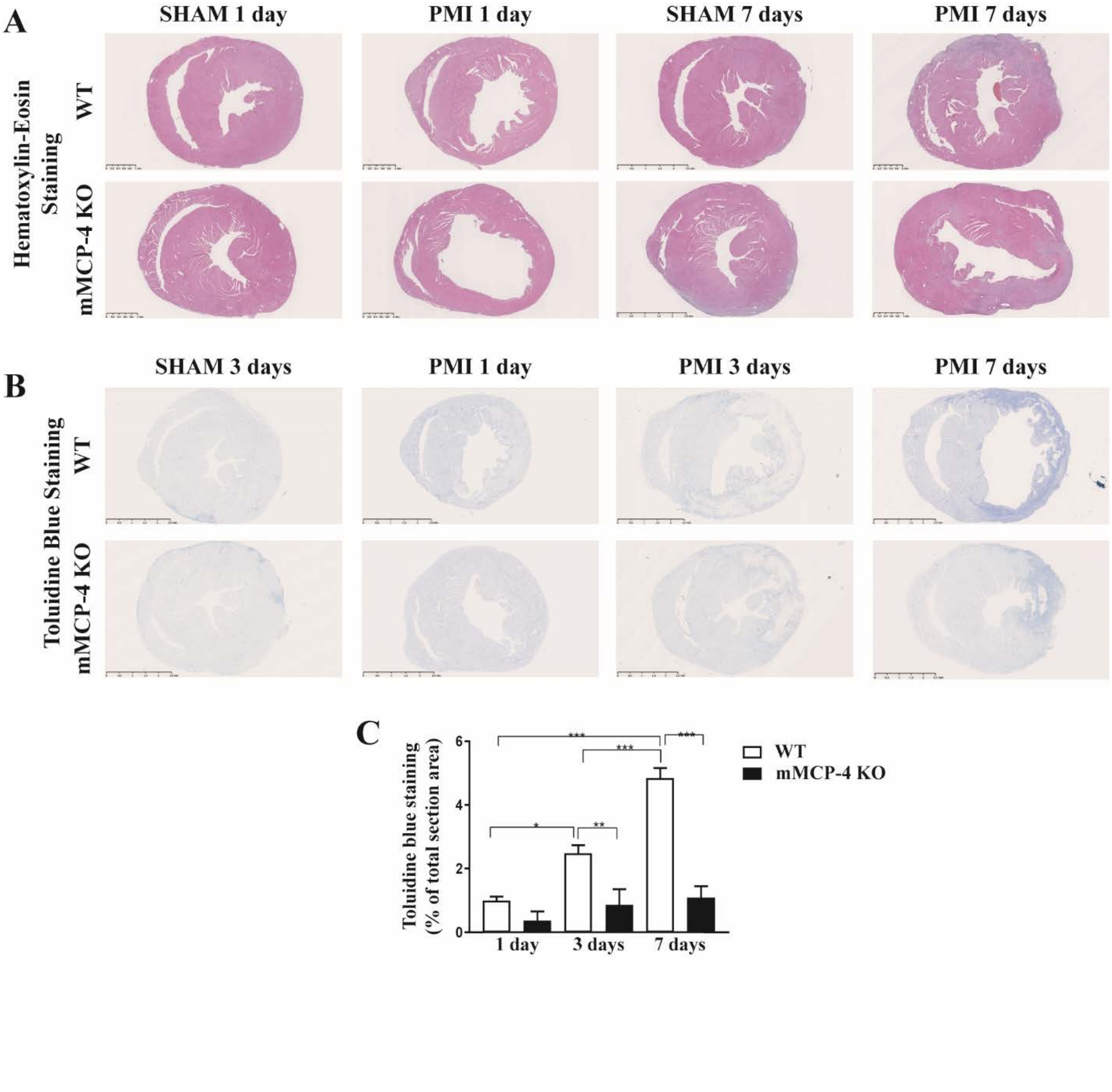

Figure S1: Hematoxyline-eosin (A) or toluidine blue (B) staining of formalin-fixed hearts from WT and mMCP-4 KO mice post-AMI. H&E staining revealed little change in heart morphology in SHAM animals, while PMI led to thinning of the left free ventricular wall with few invading cells at 1 day, with some recovery in wall thickness and large cell invasion in the infarcted area at 7 days post-PMI. Few mast cells were detected in the infarcted regions of the hearts (B), but

quantification of toluidine blue staining revealed an increase of cells in WT mice compared to mMCP-4 KO animals following PMI, with statistical significance beginning at 3 days post-PMI.

Table S1

Excel File with the 999 glycoprotein hits, data normalised with standard deviation.
